# Identification of a Novel Curcumin Derivative Influencing Notch Pathway and DNA Damage as a Potential Therapeutic Agent in T-ALL

**DOI:** 10.3390/cancers14235772

**Published:** 2022-11-24

**Authors:** Nadezda Zhdanovskaya, Sara Lazzari, Diego Caprioglio, Mariarosaria Firrincieli, Chiara Maioli, Eleonora Pace, Daniela Imperio, Claudio Talora, Diana Bellavia, Saula Checquolo, Mattia Mori, Isabella Screpanti, Alberto Minassi, Rocco Palermo

**Affiliations:** 1 Department of Molecular Medicine, Sapienza Università di Roma, 00161 Rome, Italy; 2 Department of Pharmaceutical Sciences, University of Piemonte Orientale, 28100 Novara, Italy; 3 Department of Medico-Surgical Sciences and Biotechnology, Sapienza Università di Roma, 04100 Latina, Italy; 4 Department of Biotechnology, Chemistry and Pharmacy, University of Siena, 53100 Siena, Italy

**Keywords:** curcumin, curcuminoids, T-ALL, leukemia, Notch signaling, cancer, drug discovery, anti-cancer therapy, natural products, DNA damage, inhibitors

## Abstract

**Simple Summary:**

The overall survival rate in T-cell acute lymphoblastic leukemia (T-ALL) patients is relatively high. However, the therapeutic options for refractory and relapsed cases with poor prognosis are still limited. Therefore, novel therapies are required to improve high-risk patient outcomes. Natural products can play a crucial role in developing novel drug candidates. The proven preclinical efficacy of the phytochemical compound curcumin, mediated through modulation of multiple molecular targets, including Notch signaling, is well known. However, limited data are available regarding its anti-cancer effects and its relationship with Notch in T-ALL. Our study provides evidence that curcumin reduces Notch activity and exerts anti-cancer effects in T-ALL cells by favouring DNA damage-dependent cell death. Furthermore, we identified the curcumin derivative CD2066, which is endowed with potentiated anti-growth, anti-Notch, and DNA-damaging activities. Although CD2066 anti-leukemia activity requires further investigation, our study highlights the potential of curcumin-based bioactive agents for Notch-dependent leukemia treatment.

**Abstract:**

T-cell acute lymphoblastic leukemia (T-ALL) is an aggressive hematological malignancy considered curable by modern clinical management. Nevertheless, the prognosis for T-ALL high-risk cases or patients with relapsed and refractory disease is still dismal. Therefore, there is a keen interest in developing more efficient and less toxic therapeutic approaches. T-ALL pathogenesis is associated with Notch signaling alterations, making this pathway a highly promising target in the fight against T-ALL. Here, by exploring the anti-leukemic capacity of the natural polyphenol curcumin and its derivatives, we found that curcumin exposure impacts T-ALL cell line viability and decreases Notch signaling in a dose- and time-dependent fashion. However, our findings indicated that curcumin-mediated cell outcomes did not depend exclusively on Notch signaling inhibition, but might be mainly related to compound-induced DNA-damage-associated cell death. Furthermore, we identified a novel curcumin-based compound named CD2066, endowed with potentiated anti-proliferative activity in T-ALL compared to the parent molecule curcumin. At nanomolar concentrations, CD2066 antagonized Notch signaling, favored DNA damage, and acted synergistically with the CDK1 inhibitor Ro3306 in T-ALL cells, thus representing a promising novel candidate for developing therapeutic agents against Notch-dependent T-ALL.

## 1. Introduction

T-cell acute lymphoblastic leukemia (T-ALL) is a devastating blood cancer more common in children than adults, accounting for about 20% of all acute lymphoblastic leukemia (ALL) cases. T-ALL is clinically characterized by hyper-leukocytosis, hematopoietic system failure, and medullar and extra-medullar infiltration, including frequent central nervous system (CNS) involvement [1]. Due to the introduction of intensive multi-agent chemotherapy regimens in front-line settings, T-ALL patients’ outcomes have significantly improved, with a cure rate of about 80%, though substantially higher in children than in adults [2]. However, disease relapse occurs in around 20–50% of cases, and up to 20% of patients do not respond to the first-line treatment [3]. Generally, these patients have very poor prognosis, as they have limited salvage therapeutic options besides conventional cytotoxic chemotherapies and allogeneic hematopoietic stem cell transplantation [4,5,6]. Salvage therapy is associated with acute and long-term exhausting and life-threatening side-effects, affecting survivors’ quality of life [7]. Therefore, current anti-leukemia research aims to design more effective and selective therapeutic agents and to develop novel chemo-sensitizers to potentiate the efficacy of conventional approaches [7]. 

Notably, the short cell-to-cell communication system of Notch signaling plays a prominent role in normal intrathymic T-lymphocyte development, and its aberrant activity is linked to T-ALL pathogenesis and progression [8,9]. Canonical Notch signaling is orchestrated by the interaction between evolutionarily conserved trans-membrane receptors (Notch1–4) and ligands (Jagged-1, -2, Delta-like-1, -3, -4). In T-ALL, gain-of-function mutations occurring at the NOTCH1 gene are found in about 50–60% of patients [10], and high expression of the NOTCH3 gene can frequently be observed in patients’ samples analyzed [11]. In addition, over-activation of signaling has been reported in about 15% of cases of T-ALL harboring inactivating mutations in the Notch negative regulator FBXW7 [12]. Aberrant Notch activity strengthens the transcription of critical factors controlling T-cell survival and differentiation during physiological thymopoiesis, including MYC, NRARP, DTX1, HES1, PTCRA, and non-coding RNAs, such as miR-223 and LUNAR1, thus favoring the onset and progression of T-cell lineage leukemia [13,14]. Notably, Notch family members, independently from their transcriptional functions, interact with critical pathways in maintaining survival, differentiation, metabolism, and DNA damage repair in T-ALL [15,16,17]. 

In keeping with Notch’s oncogenic role, several approaches to inhibit its pathway activity are under exploration in pre-clinical and clinical studies [18]. However, despite proposed strategies showing high effectiveness in pre-clinical settings, their practical application is limited by low clinical efficacy and/or on target-dependent dose-limiting intestinal toxicity [18,19,20,21], leaving this field of anti-cancer research still under-explored. Recently, small molecules that interfere with the function of the Notch transcription complex have emerged as more effective and potentially safer Notch inhibitors [22,23]. 

Several naturally occurring phytochemicals are being actively investigated due to their predicted low toxicity, multi-target action, and efficacy against cancer cells and stem cells [24,25,26]. Among them, the polyphenolic compound curcumin has shown promising cancer preventive and anti-tumor properties, both as monotherapy and combined with other drugs, in various neoplastic pathologies, including leukemia [27,28,29,30,31,32,33,34]. Evidence indicates that the anti-cancer activity of curcumin and its derivatives depends on their startling intrinsic multi-target effects. Indeed, treatment with these compounds has been shown to interfere in a cell-context-dependent fashion with critical cellular networks regulating proliferation, survival, and the metabolism of cancer cells, including Notch, and DNA damage and repair machinery [35,36,37,38,39]. However, the mechanism underlying Notch suppression remains unclear and very few studies on the efficacy of the treatment in T-cell-lineage leukemia have been conducted [40,41,42]. Moreover, contradictory results have been obtained in normal and malignant T-cell contexts, indicating that curcumin might both prevent and favor DNA damage by acting as a pro- or anti-oxidant and by promoting or interfering with the DNA-repair machinery in a cell-type- or dosage-dependent manner [43,44,45,46]. 

We investigated the potential anti-leukemic capacity of curcumin by exploring to what extent it counteracts cell viability and Notch pathway activity and modulates levels of DNA damage and DNA damage response in T-ALL cell lines with distinct Notch gene mutational states. Moreover, we designed and synthesized a series of curcumin derivatives with a substituted aromatic link or linker region. The screening of these curcuminoids for strength in restraining T-ALL cell growth led to the identification and characterization of a novel compound named CD2066, endowed with potentiated anti-proliferative, anti-Notch, and DNA-damaging activities compared to the parent curcumin. Overall, CD2066 emerged as a promising novel candidate for developing bioactive and therapeutic agents against T-ALL. 

## 2. Materials and Methods

### 2.1. Chemistry

IR spectra were recorded on an Avatar 370 FT-IR Techno-Nicolet apparatus. ^1^H (400 MHz) and ^13^C (100 MHz) NMR spectra were measured on a Bruker Avance 400 MHz spectrometer. Chemical shifts were referenced to the residual solvent signal (CDCl_3_: δH = 7.25, δC = 77.0, CD_3_OD: δH = 3.34, δC = 49.0, DMSO: δH = 2.50, δC = 39.5, (CO(CD_3_)_2_: δH = 2.05, δC = 206.7, 29.9). Low- and high-resolution electrospray ionization mass spectrometry (ESI-MS) data were determined on an LTQ OrbitrapXL (Thermo Scientific, Waltham, MA, USA) mass spectrometer. Chemical reactions were monitored by thin-layer chromatography (TLC) by visualizing educts and products on Merck 60 F254 (0.25 mm) plates upon staining with 5% H_2_SO_4_ in EtOH and heating. Organic phases were dried with Na_2_SO_4_ before evaporation. Chemical reagents and solvents were purchased from Merck, TCI Europe or Fluorochem and were used without further purification unless stated otherwise. Petroleum ether with a boiling point of 40–60 °C was used. Silica gel 60 (70–230 mesh) was used for gravity column chromatography (GCC).

### 2.2. Synthesis of (1E,4Z,6E)-5-Hydroxy-1,7-bis(4-hydroxy-2-methylphenyl)hepta-1,4,6-trien-3-one (CD2061)

To a stirred solution of 4-hydroxy-2-methylbenzaldehyde (1) (2 eq. mol) in dry DMF (2 mL/mmol), acetylacetone (2) (1 eq. mol), B_2_O_3_ (1.6 eq. mol), B(OCH_3_)_3_ (0.73 eq. mol) and n-butyl amine (0.08 eq. mol) were sequentially added. The solution was stirred at 40 °C until substantial conversion was observed by TLC, then quenched with H_2_SO_4_ (2M) and extracted with EtOAc. The organic layers were dried over Na_2_SO_4_, filtered, and evaporated. Compound CD2061 was obtained after purification by GCC on silica gel in 50% yield. ^1^H NMR (400 MHz, (CO(CD_3_)_2_) δ: 7.90 (d, J = 15.6 Hz, 2H), 7.64 (d, J = 9.2 Hz, 2H), 6.76 (m, 4H), 6.63 (d, J = 15.6 Hz, 2H), 6.01 (s, 1H), 2.41 (s, 6H); ^13^C NMR (100 MHz, CO(CD_3_)_2_) δ: 183.70, 159.33, 140.01, 137.20, 128.12, 125.37, 121.90, 121.87, 117.38, 113.84, 101.14, 19.00. HRESIMS *m*/*z* [M+H]^+^ 337.14313 (calcd. for C_21_H_21_O_4_, 337.14344).

### 2.3. Synthesis of 4,4’-((1E,1’E)-(1H-Pyrazole-3,5-diyl)bis(ethene-2,1-diyl))bis(3-methylphenol) (CD2066)

To a stirred solution of compound 3 (1 eq. mol) in acetic acid (2.5 mL/1 mmol), hydrazine monohydrate (2.3 eq. mol) was added. The reaction was stirred at 50 °C overnight, quenched with the addition of brine and extracted with EtOAc. The organic layers were dried over Na_2_SO_4_, filtered, and evaporated. The residue was purified by GCC on silica gel furnishing CD2066 in 75% yield. ^1^H NMR (400 MHz, CO(CD_3_)_2_) δ: 7.52 (d, J = 1.2 Hz, 2H), 7.39 (d, J = 16.4 Hz, 2H), 6.90 (d, J = 16.4 Hz, 2H), 6.74 (d, J = 3.0 Hz, 2H), 6.71 (s, 3H), 2.36 (s, 6H); ^13^C NMR (100 MHz, CO(CD_3_)_2_) δ: 157.23, 137.29, 127.45, 127.00, 126.34, 117.02, 116.80, 113.45, 19.13. HRESIMS *m*/*z* [M+H]^+^ 333.15946 (calcd. for C_21_H_21_N_2_O_2_, 333.15975).

### 2.4. Cell Lines and Treatments

KOPT-K1 [47,48], DND41 [10,48] and U937 [49,50] cells were cultured in RPMI-1640 (21875091; Gibco, Carlsbad, CA, USA), supplemented with 10% fetal bovine serum (FBS) (10270106; Gibco), 1 nmol/L L- glutamine (G7513-100ML; Sigma-Aldrich, St. Louis, MO, USA) and 10 nmol/L penicillin/streptomycin (P0781-100ML; Sigma-Aldrich), while TALL1 [48,51], Loucy [52,53] and THP1 [50,54] cells were maintained in RPMI-1640 (21875091; Gibco) containing 15% FBS (10270106; Gibco), 1 nmol/L L-glutamine (G7513-100ML; Sigma-Aldrich) and 10 nmol/L penicillin/streptomycin (P0781-100ML; Sigma-Aldrich). Cell line details are provided in the database Cellosaurus (https://www.cellosaurus.org, accessed on 10 October 2022), respectively, with the following accession numbers: KOPT-K1 (CVCL_4965), DND41 (CVCL_2022), TALL1 (CVCL_1736), U937 (CVCL_0007), THP1 (CVCL_0006) and Loucy (CVCL_1380). Specifically, KOPT-K1, DND41 and TALL1 cells were provided by Dr. Stefano Indraccolo (Istituto Oncologico Veneto IOV—IRCCS, Padova, Italy). The Loucy cell line was obtained from the American Type Culture Collection (CRL-2629; ATCC, Manassas, VA, USA). U937 and THP1 cell lines were provided by Dr. Francesco Fazi (Sapienza University, Rome, Italy). All the above-mentioned cells were cultured at 37 °C and 5% CO_2_, tested for mycoplasma contamination using a PCR detection kit (G238; Abm Inc., Richmond, Canada), and treated with curcumin [55], Ro3306 (SML0569; Sigma-Aldrich) and the seventeen synthetic molecules for the indicated concentrations and times of exposure. 

### 2.5. Cell Viability Assay and IC_50_ Determination

KOPT-K1, DND41, TALL1 and Loucy cells were seeded in a 24-well flat-bottom plate at 5 × 10^5^ cells/mL, while U937 and THP1 cells were seeded at 2 × 10^5^ cells/mL and then treated at the indicated times and compound concentrations. The number of viable and dead cells was counted by a Trypan Blue exclusion assay (T8154; Sigma-Aldrich) and normalized to the number of cells treated with the solvent DMSO alone (D5879; Sigma-Aldrich). Relative cell viability values were further fitted by a non-linear regression model to calculate the absolute IC_50_ of the abovementioned compounds for each cell line using GraphPad Prism 8.0 software (GraphPad Software, San Diego, CA, USA).

### 2.6. Protein Extracts Preparation, Antibodies and Western Blot

Cells were lysed in Laemmli buffer (1610737; Biorad, Hercules, CA, USA) by sonication and centrifuged at 13,000 rpm at 4 °C for 15–20 min to remove debris. Protein concentrations of whole cell lysates were determined by spectrophotometric analysis using the dye reagent Bradford (5000006; Biorad). Before immunoblotting, samples were added with β-mercaptoethanol (M6250; Sigma-Aldrich) and boiled for 10 min at 100 °C. Protein extracts were run on SDS-polyacrylamide gels and then transferred to nitrocellulose membranes (1620115; Biorad). Next, the membranes were blocked at RT for 1 h with bovine serum albumin (A2153; Sigma-Aldrich) in 5% TBS/tween 0.1% and then incubated at 4 °C overnight with antibodies against: Notch1Val1744 (4147; Cell Signaling Technology, Danvers, MA, USA), Notch1 (D1E11) (3608; Cell Signaling Technology), Notch3 (2889; Cell Signaling Technology), PARP (9542; Cell Signaling Technology), p27 Kip1 (D37H1) (3688; Cell Signaling Technology), pcdc2 Tyr15 (10A11) (4539; Cell Signaling Technology), cdc2 (77055; Cell Signaling Technology), Phospho-Akt (Ser473) (9271; Cell Signaling Technology), Akt (pan) (40D4) (2920; Cell Signaling Technology), Hes1 (D6P2U) (11988; Cell Signaling Technology), Phospho-Histone H2A.X (Ser139) (20E3) (9718; Cell Signaling Technology), Histone H2A.X (D17A3) (7631; Cell Signaling Technology), ATM (D2E2) (2873; Cell Signaling Technology), ATM phospho S1981 (EP1890Y) (ab81292; Abcam, Cambridge, UK) and β-actin (A5441; Sigma-Aldrich), followed by hybridization with antibodies, HRP conjugated anti-rabbit (A120-108P; Bethyl Laboratories, TX, USA) or anti-mouse (A90-116P; Bethyl Laboratories, TX, USA). Immunoreactions were developed by enhanced chemiluminescence with Clarity™ Western ECL Substrate (170-5061; Biorad); immunoreactive bands were visualized using the c300 imaging system (Azure Biosystems, Sierra Court, CA, USA) and then quantified through ImageJ analysis software.

### 2.7. Gene Expression Analysis by Real-Time RT-PCR

Total RNA was extracted from cells using TRIZOL reagent (15596018; Invitrogen, Carlsbad, CA, USA) and reverse-transcribed using a high-capacity cDNA reverse-transcription kit (4368814; Applied Biosystems, Foster City, CA, USA), according to the manufacturer’s protocol. Taqman Gene Expression Master Mix (4440047) and Taqman Gene Expression Assays for NOTCH1 (Hs01062014_m1), NOTCH3 (Hs00166432_m1), DELTEX1 (Hs01092201_m1), HES1 (Hs00172878_m1), and 18S (4352930E) were purchased from Applied Biosystems. Relative gene expression for IER5, BAP1, FEN1, RNF8, RAD51 and PCNA was determined by SYBR green Q-PCR using a SensiFAST SYBR Hi-ROX Kit (BIO-92020; Bioline, London, UK) and using the primers described in Table S1. PCR reactions were run on a MicroAmp ^TM^ Fast 96-well reaction plate, 0.1 mL (4346907; Applied Biosystems) at 95 °C for 10 min, followed by 40 cycles at 95 °C for 15 s and 60 °C for 30 s, and were performed using the StepOne™ Real-Time PCR System (4376592; Applied Biosystems). Relative quantification was carried out using the comparative ΔΔCT method [56]. 18S ribosomal RNA gene (18S rRNA) expression was used to normalize mRNA levels. Measurements were performed in technical triplicates; the figures show the averages ±SD of at least three biological replicates.

### 2.8. Cell-Cycle Analysis and Apoptosis Detection

KOPT-K1, DND41, and TALL1 cells were seeded at 5 × 10^5^ cells/mL and treated for 48 h with indicated compound concentrations. For cell-cycle analysis, cells were washed with PBS, fixed in chilled 70% ethanol at 4 °C for at least 30 min, and treated with RNase A (10 mg/mL) (EN0531; ThermoFisher Scientific, Bremen, Germany) for 15 min at 37 °C and then stained with 10 µL of propidium iodide solution (1 mg/mL) (P3566; Invitrogen) at room temperature. Fluorescence-activated cell-sorting (FACS) analysis was carried out using FACS-Calibur (BD Biosciences, San Jose, CA, USA), and data analysis was performed with CellQuestPro software (BD Biosciences). Caspase 3 and 7 activity levels were assessed by luminescence assay (G8091, Caspase-Glo^®^ 3/7 assay system; Promega Corporation, Madison, WI, USA), according to the manufacturer’s instructions. Data acquisition was carried out using the GloMax Multidetection System (Promega Corporation).

### 2.9. Comet Assay

An alkaline comet assay was conducted as described in [57], with minor modifications. Briefly, cell suspensions were mixed with 0.75% agarose (161-3102; Biorad,) (at 37 °C) at a ratio of 1:10 (*v*/*v*) and immediately transferred on a microscope slide pre-coated with 1% agarose (161-3102; Biorad). After solidification, slides were immersed in pre-chilled lysis buffer (2.5M NaCl (S3014-5KG; Sigma-Aldrich), 100 mM EDTA (161-0729; Biorad), 10 mM Trizma (T1503-1KG; Sigma-Aldrich), 10% DMSO (D5879-100ML, Sigma-Aldrich), 1% Triton-100 (T8787-50ML; Sigma-Aldrich) and 200 mM NaOH (S8045; Sigma-Aldrich), pH 10) for 45 min to 1 h at 4 °C in the dark, depending on the cell line. After lysis, the slides were immersed in pre-chilled alkaline buffer (300 mM NaOH (S8045; Sigma-Aldrich) and 1 mM EDTA (161-0729; Biorad), pH > 13) for 30 min at 4 °C in the dark to allow DNA unwinding. Slides were then placed on a horizontal gel electrophoresis chamber (1704468; Biorad) filled with cold alkaline electrophoresis solution (300 mM NaOH (S8045; Sigma-Aldrich) and 1 mM EDTA (161-0729; Biorad), pH > 13. Electrophoresis was carried out for 30 min in the dark at fixed voltage and amperage (1 V/cm, 300 mA). After migration, the slides were rinsed with water, fixed for 5 min in 70% ethanol and stained with Vista Green DNA Dye 10000X (ab238544; Abcam) for 15 min at RT in the dark, according to the manufacturer’s recommendations. Images were acquired through a fluorescence microscope (Leica Microsystems, Milan, Italy) using a GFP filter. The tail moment was measured using OpenComet Software (an automated tool for comet assay image analysis [58]. Approximately 100 cells/sample were counted; the figures show the averages ± SD of at least three independent experiments.

### 2.10. Rescue Assays

Growth susceptibility to the exposure to increasing dosages of curcumin and CD2066 was compared between DND41 cells transduced with a retroviral construct encoding the entire murine Notch1 intracellular fragment CMMP-ICN1-IRES-EGFP (mICN1) and the DND41 counterpart cells transduced with the relative empty control vector CMMP-IRES-EGFP (empty), as previously described [59]. Cells were seeded in a 24-well flat-bottom plate at 5 × 10^5^ cells/mL and then exposed to the indicated compound concentrations for 48 h. The number of viable cells was analyzed using a Trypan Blue exclusion assay (T8154; Sigma-Aldrich) and normalized to the number of the counterpart cells treated with the vehicle DMSO (D5879; Sigma-Aldrich).

### 2.11. Drug Interaction Analysis

Both the Bliss independence model and combination index analysis based on the Loewe additivity principle (for each dose of drug A exists an equal dose of drug B which gives the same effect) are commonly used for evaluating drug combination effects. The Bliss independence model states that treatment outcomes result from stochastic processes and presupposes that the expected combined effects of two drugs are the product of their individual effects, based on the hypothesis that each drug acts by independent mechanisms occurring simultaneously and mutually non-exclusively, and contribute to a common outcome without interfering with one another [60]. The model is based on a quantitative measure called “excess over Bliss” which represents the difference between the observed combined effect of the two drugs and the expected combined effect. The expected combination effect is calculated using the equation Effect (a + b) = E(a) + E(b) − E(a) × E(b). Positive and negative values of excess over Bliss indicate synergistic and antagonistic interaction, respectively, whereas null values indicate additive effects. In addition, the observed combination effect can be compared to the expected combination effect by analysing the combination index, which can be determined as the ratio between the expected combination effect, calculated as described above, and the observed combination effect. CI values = 1 are considered additive, while CI < 1 and CI > 1 are considered synergistic or antagonistic, respectively [60].

### 2.12. Statistical Analysis

All statistical tests were carried out using GraphPad Prism version 8.0 (GraphPad Software, San Diego, CA, USA). Statistical data analysis between two groups was carried out using two-tailed Student’s paired and unpaired *t*-tests. Multiple comparisons analysis was carried out by one-way ANOVA followed by Tukey or Sidak post hoc tests. Differences were considered significant when *p*-values < 0.05. Values significance: * *p* < 0.05, ** *p* < 0.01, *** *p* < 0.001, **** *p* < 0.0001. 

## 3. Results

### 3.1. Curcumin Antiviability Effects in T-ALL Cells Combine with Notch Suppression and DNA Damage Accumulation

Evidence from preclinical studies supports curcumin’s anticancer activity in different types of solid and hematological tumors, and clinical investigations have prompted its evaluation for therapeutic efficacy, safety, and tolerability as a monotherapy or in combination with other drugs in patients with different diseases, including cancers [61]. Among the multitude of its molecular target pathways, curcumin is known to interfere with Notch signaling transduction in cell models of solid cancers, thus suggesting its interaction with this pathway [35,36,38,39]. Nonetheless, the effectiveness of curcumin on Notch is under-explored in T-ALL; its anti-leukemic activity has mostly been linked to mitochondrial Ca2+ overload, ROS induction, and (PI3K)/AKT pathway inhibition that lead to cell cycle disruption and apoptotic cell death induction [40,41,62,63]. Here, we investigated the effects of curcumin on the viability of three human T-ALL cell lines with different statuses of Notch receptor activation: KOPT-K1 and DND41 cells harboring constitutive ligand-independent activation of the signaling due to mutations of HD and PEST domains of the Notch1 receptor [10], and TALL1 cells exhibiting Notch over-activation due to gain-of-function mutations involving the HD domain of the Notch3 receptor [64]. In addition, KOPT-K1 and DND41 express the wild-type (WT) gene of NOTCH3, whereas TALL1 cells possess a not-activated WT form of the Notch1 receptor. Cell sensitivity to curcumin was estimated as half-maximal growth inhibitory concentration (IC_50_), determined using non-linear regression analysis of growth inhibition curves obtained after 48 h (abbreviation: h) of treatment with increasing doses of the compound (0, 0.5 μM, 1 μM, 2.5 μM, 5 μM, 10 μM, 20 μM, 50 μM) in KOPT-K1 and TALL1 and (0, 0.5 μM, 2.5 μM, 7.5 μM, 10 μM, 25 μM, 50 μM, 100 μM) in DND41. Through this approach, we demonstrated that curcumin exerts antiviability effects in T-ALL cells at low micromolar doses, determined as IC_50_ in the concentration range between 6.33 μM and 13.255 μM (Table 1 and Figure S1).

**Figure 1:**
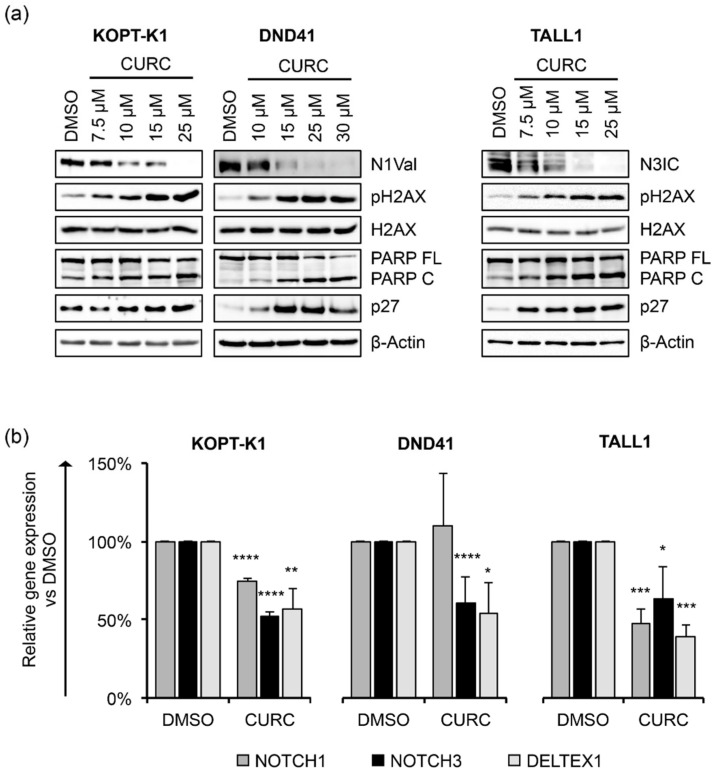
Curcumin decreases Notch activation and modulates cell cycle progression and apoptotic regulators in a dose-dependent manner in T-ALL cell lines. (**a**) Levels of N1Val, N3IC, pH2AX, H2AX, full-length PARP protein (PARP FL), cleaved form of PARP (PARP C), and p27 after 48 h of treatment with the indicated concentrations of curcumin (CURC) or the vehicle alone (DMSO). β-Actin was used as a loading control. Uncropped Western blots and the relative quantification of proteins levels related to this figure are displayed in Figure S2. (**b**) Relative NOTCH1, NOTCH3, and DELTEX1 gene expression levels normalized to 18S rRNA following 48 h of exposure to DMSO or CURC in KOPT-K1 (10 µM), DND41 (15 µM) and TALL1 (10 µM). Histograms show the mean of results obtained from 3 independent experiments ± standard deviation (SD). Statistical significance was assessed using unpaired *t*-test. *p* values * *p* < 0.05, ** *p* < 0.01, *** *p* < 0.001, **** *p* < 0.0001.

We next explored curcumin’s interaction with Notch signaling and other critical molecular pathways in dose-response assays. To this end, upon 48 h of cell exposure to increasing doses of the compound, we investigated the expression of the activated domain of Notch1 (N1Val) by Western blot using an antibody against valine 1744 and the Notch3 intracellular domain (N3IC), and the phosphorylation status of H2AX at serine 139 (pH2AX), the accumulation of which represents an early event necessary for the DDR. In addition, we evaluated the cleavage of poly-ADP-ribose polymerase (PARP) occurring during caspase-mediated cell death and the expression of the negative regulator of the cell cycle progression p27kip1 (p27). We detected decreased expression of N1Val in KOPT-K1 and DND41 cells and N3IC in TALL1 cells and observed increased levels of pH2AX and cleavage of PARP in a dose-dependent manner at micromolar concentrations around the respective IC_50_s, starting from the minimal dosage of the compound in the three T-ALL cell lines (Figure 1a).

**Figure 2:**
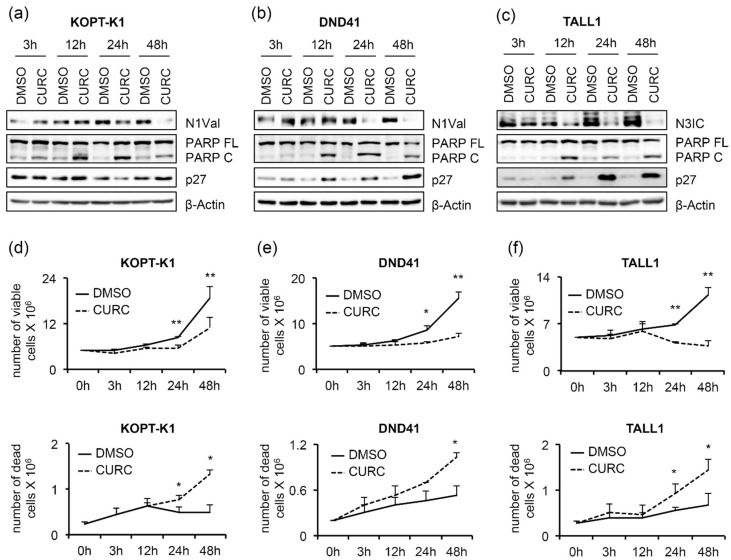
Antiviability and pro-apoptotic effects of curcumin are associated with Notch signaling inhibition in T-ALL cells. T-ALL cells were treated for 48 h with CURC or DMSO at the indicated concentrations: 10 μM for KOPT-K1 and TALL1, or 15 μM for DND41. Protein extracts were collected at 3, 12, 24, and 48 h and analyzed by Western blotting. Protein expression of N1Val in (**a**) KOPT-K1 and in (**b**) DND41, and of N3IC in (**c**) TALL1 cells. Levels of PARP FL, PARP C, and p27 in (**a**) KOPT-K1, (**b**) DND41, and (**c**) TALL1 cell lines at indicated time points. β–Actin was used as a loading control. Uncropped Western blots and the relative quantification of proteins levels related to this figure are displayed in Figure S3. Histograms show the number of viable (upper panels) and dead (lower panels) in (**d**) KOPT-K1, (**e**) DND41, and (**f**) TALL1 cells at different time points evaluated through the Trypan Blue exclusion assay. Data represent the mean value of three independent experiments ± SD. Statistical significance was calculated using a paired *t*-test. *p* values, * *p* < 0.05, ** *p* < 0.01.

Furthermore, curcumin exposure promoted p27 expression starting from concentrations close to the IC_50_s in DND41 and TALL1 and slightly above the IC_50_ in KOPT-K1 cells (Figure 1a). Of note, functional repression of the Notch pathway was further confirmed by the transcriptional inhibition of the Notch target gene DELTEX1, and both the receptors NOTCH1 and NOTCH3 upon exposure to curcumin in the T-ALL cells, except for NOTCH1 in DND41, which did not undergo significant alterations (Figure 1b). To corroborate curcumin’s effectiveness on T-ALL cell growth and its dynamic interaction with the above-described pathways, we performed time-course experiments by treating KOPT-K1 and TALL1 cells with 10 µM and DND41 with 15 µM of curcumin. As a result, we observed declined levels of expression of N1Val in KOPT-K1 and DND41 cells (Figure 2a,b) and N3IC in TALL1 cells (Figure 2c), starting from 24 h and 12 h of treatment, respectively.

**Figure 3:**
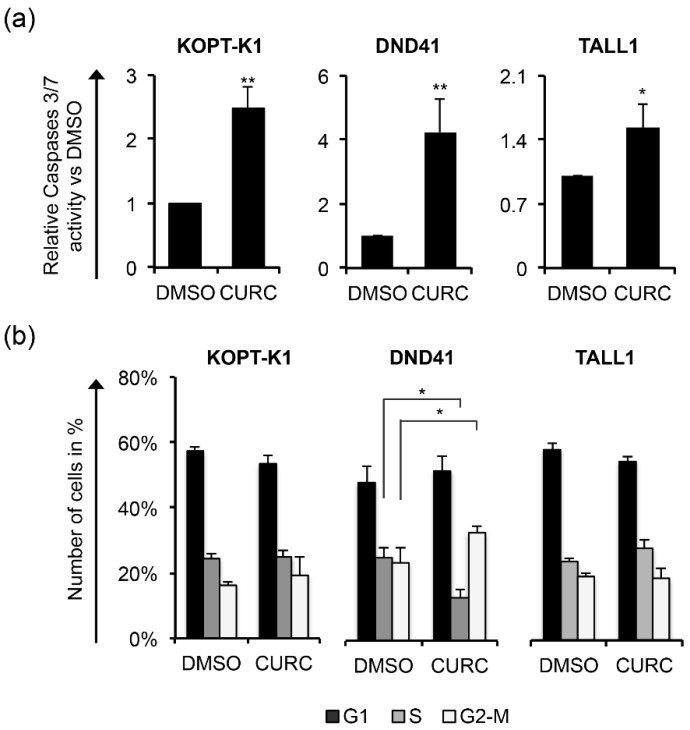
Curcumin possesses pro-apoptotic activity in T-ALL. T-ALL cells were treated for 48 h with CURC or DMSO at the indicated concentrations: 10 μM for KOPT-K1 and TALL1 or 15 μM for DND41. (**a**) Apoptosis was assessed by monitoring the activation of caspase 3 and 7. (**b**) Cell distribution along cell-cycle phases was investigated by flow cytometry analysis of the DNA content after propidium iodide (PI) staining. The bars represent the mean of three independent experiments ± SD. Statistical significance was calculated using unpaired *t*-test and paired *t*-test for caspase 3/7 activity and cell-cycle distribution, respectively. *p* values refer to the comparison between curcumin and DMSO samples: * *p* < 0.05, ** *p* < 0.01. Representative images of the cell-cycle analysis are shown in Figure S4.

Increased PARP cleavage and p27 expression preceded or occurred simultaneously with Notch inhibition, as their modulations were noticeable after 12 h of exposure to the compound in all cell lines analyzed, except for p27 in KOPT-K1, the level of which slightly increased at 48 h (Figure 2a–c). From the kinetic standpoint, the curcumin impact on cell viability and cell death, detected by Trypan Blue exclusion assay, was combined with and/or followed the above-described molecular events (Figure 2d–f), thus suggesting that curcumin’s mechanism of action might interfere with Notch modulation in leukemic cells. To further verify the curcumin properties, we determined the apoptotic rate and cell distribution within the phases of the cycle following the compound administration. Consistent with the cleavage of PARP, 48 h of curcumin exposure to around-IC_50_s concentrations increased the activity of caspases 3 and 7 in the T-ALL cell lines (Figure 3a). 

**Figure 4:**
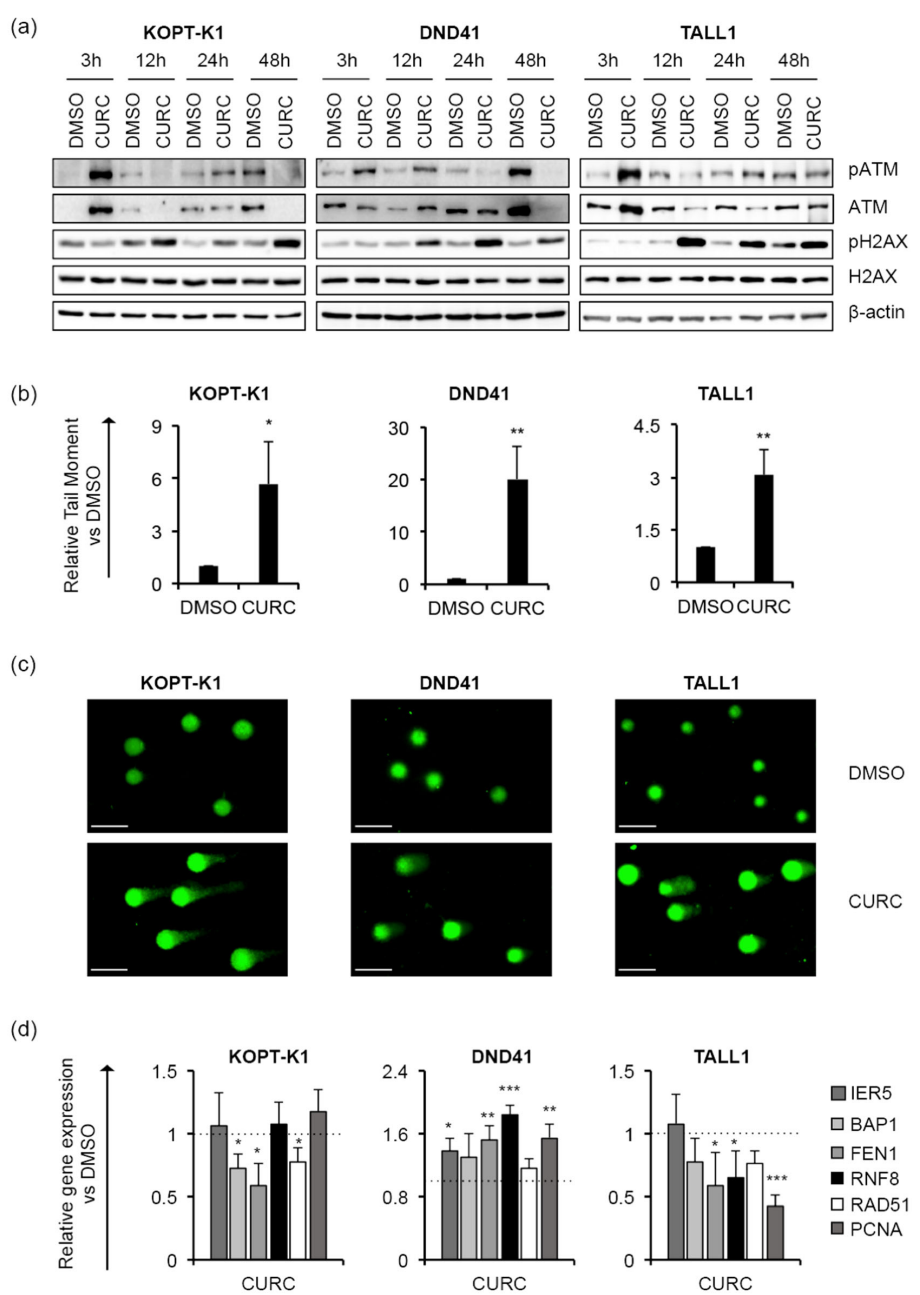
Curcumin favours DNA damage in T-ALL cells. T-ALL cells were treated with CURC or DMSO at the indicated concentrations: 10 μM for KOPT-K1 and TALL1, or 15 μM for DND41. (**a**) Levels of ATM phosphorylation at serine 1981 (pATM), ATM expression (ATM), pH2AX and H2AX in KOPT-K1, DND41, and TALL1 cells at indicated time points. β–actin was used as a loading control. Uncropped Western blots and the relative quantification of proteins levels related to this figure are displayed in Figure S5. (**b**) Relative quantification of the comet tail’s moment in KOPT-K1, DND41, and TALL1 at 24 h of treatment with curcumin. Data represent the mean value of three independent experiments ± SD. Statistical significance was calculated using an unpaired *t*-test. *p* values, * *p* < 0.05, ** *p* < 0.01. (**c**) Representative images of DNA comets in KOPT-K1, DND41, and TALL1 cells from curcumin treatments. Scale bar: 100 μm (**d**) Relative gene expression levels of IER5, BAP1, FEN1, RNF8, RAD51, and PCNA at 48 h of exposure to CURC versus control cells (DMSO). 18S rRNA was used as a loading control. Histograms show the mean results obtained from three independent experiments ± SD. Statistical significance was assessed using an unpaired *t*-test, * *p* < 0.05, ** *p* < 0.01, *** *p* < 0.001.

Although 48 h of treatment with curcumin modulated the cell-cycle regulator p27, it did not alter the cell-cycle profile in KOPT-K1 and TALL1. Conversely, it significantly decreased DND41 cells in the S phase from 25% to 13% and increased the cell percentage in the G2/M compared with the counterpart treated with the vehicle alone from 23% to 33% (Figure 3b). Overall, our observations indicated that curcumin acts mainly as a cytotoxic drug by corrupting cell-cycle progression and promoting programmed cell death. Consistent with previous studies reporting curcumin and DDR pathway interaction [46], we found that 3 h of treatment increased the expression levels and upregulated the ser-1981 phosphorylation of the DDR central regulator ATM. Accordingly, 12 h of exposure resulted in the accumulation of the ATM target substrate pH2AX, further indicating that it rapidly promoted DNA damage in T-ALL (Figure 4a). 

In addition, by single-cell gel electrophoresis assay (known as a comet assay), we identified increased DNA fragmentation in the T-ALL cells exposed to curcumin compared to the counterpart cells treated with the vehicle alone, with comet tail moment values more prolonged by 3 to 20 times than the control cells (Figure 4b,c). Nevertheless, the treatment reduced the steady-state transcriptional levels of critical DDR-machinery-related components, including BAP1, FEN1 and RAD51 in KOPT-K1 and FEN1, RNF8 and PCNA in TALL1 cells (Figure 4d), thus suggesting that curcumin could compromise DDR activity. Conversely, in DND41, curcumin promoted the expression of all the DDR factors examined, except for BAP1 and RAD51. Overall, our results indicate that the anti-cancer action of curcumin in T-ALL combines with Notch signaling suppression and DNA damage accumulation and suggest that it might promote DNA damage-dependent cell death by impairing DDR activity.

### 3.2. Design and Synthesis of Novel Curcumin Derivatives

**Figure 5:**
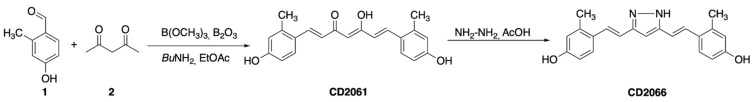
General strategy for the synthesis of alkylated curcuminoids. Synthesis of CD2061 and CD2066 as an example.

The therapeutic efficacy of curcumin in clinical applications is limited by poor bioavailability and efficacy. Therefore, several researchers have sought to improve its effectiveness and pharmacological properties using different approaches, including developing novel curcumin-based compounds [65,66,67]. We contributed to this field by aiming to dissect the pharmacophore of curcumin, synthesizing new derivatives with different decoration patterns of the aromatic rings, and by reducing the length and changing the nature of the functional groups of the heptanoid chain of the natural dye [55,68,69,70]. Taken together, the results of our previous studies suggest that a reduction in conformational mobility represents a possible strategy to obtain more potent and more selective curcumin-based derivatives. Curcumin is characterized by a complex conformational equilibrium and, to simplify it, we designed a class of constrained curcuminoids, where the intrinsic flexibility of the natural dye is reduced by the insertion of alkyl groups both on the aromatic moieties and the heptanoid chain [71]. This maneuver allowed us to obtain three different small libraries of mono- and poly-alkylated compounds (Figure S6) that were synthesized under Pabon conditions to obtain seventeen curcuminoid analogues that, after treatment with hydrazine in acetic acid, afforded the corresponding pyrazoles (Figure 5).

### 3.3. Identification of a Novel Curcumin Analog with Antiviability Activity against T-ALL Lines

**Figure 6:**
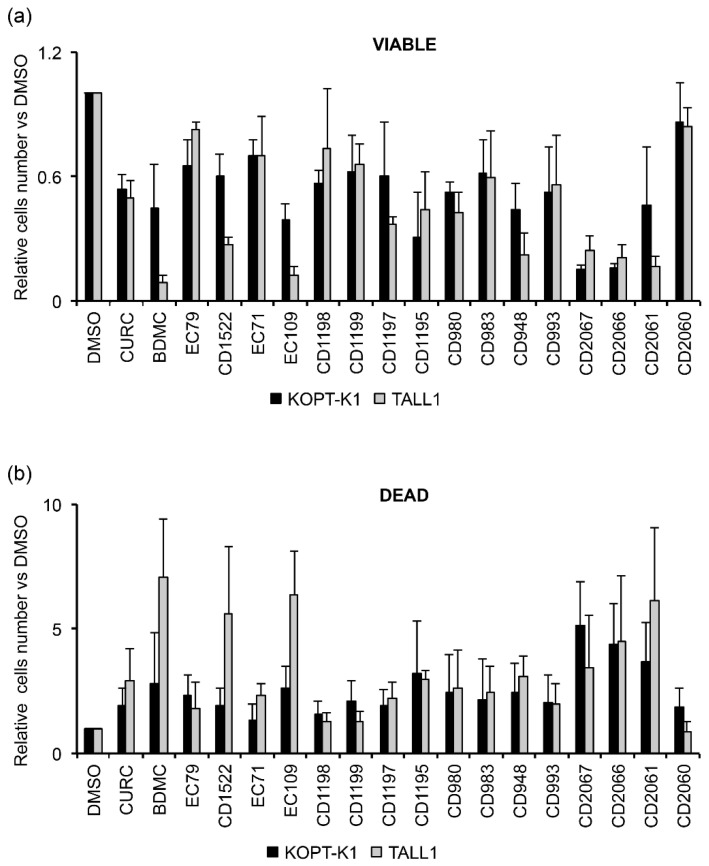
Biological screening of seventeen curcumin derivatives compared to curcumin in KOPT-K1 and TALL1 cells. Histograms show the mean of four independent experiments ± SD of the relative number of (**a**) viable or (**b**) dead cells following treatment with 7.5 μM of indicated compounds or DMSO for 48 h and measured by Trypan Blue assay.

To evaluate the effectiveness of the seventeen novel curcuminoids (Figure S6), we investigated their growth-inhibitory potency in Notch1-dependent KOPT-K1 and Notch3-dependent TALL1 cells, in which curcumin inhibited cell growth with IC_50_s of 8.2 μM and 6.3 μM, respectively. Therefore, we treated the cell lines with 7.5 μM of each compound for 48 h and compared their anti-growth potency by Trypan Blue exclusion assay. We set up a 50% decrease in cell viability as a threshold value for selecting the molecules worthy of further investigation. This screening allowed us to identify the compounds EC109, CD2066, and CD2067 as the most effective in both cell lines. Therefore, we excluded the compounds EC79, EC71, CD1198, CD1199, CD1197, CD1195, CD980, CD983, CD993, and CD2060 from our study as they showed weaker or comparable anti-growth potential concerning the scaffold of origin. In addition, we considered the molecules BDMC, CD1522, CD948, and CD2061 unworthy of further analysis, as they were more effective than curcumin only in TALL1 cells (Figure 6a).

Of note, the selected compounds also increased the percentage of cell death in both T-ALL cell lines (Figure 6b). However, by investigating the anti-growth potency of the chosen molecules in dose-response assays, we identified CD2066 as the most active compound of the series in terms of inhibiting T-cell viability, with IC_50_ values in the low nanomolar range (between 0.032 μM and 0.060 μM) and with a power of action about one hundred times stronger than curcumin. On the other hand, the molecules CD2067 and EC109 exhibited lower effectiveness, with IC_50_ values close to the reference compound curcumin ranging from 1.471 to 7.757 μM (Table 2 and Figure S7).

Notably, CD2066 showed comparable growth inhibitory activity in DND41 cells with IC_50_ values of 0.100 ± 0.014 μM (Figure S8).

### 3.4. CD2066 Interferes with Notch Signaling Activity and Counteracts T-ALL Cell Line Viability

**Figure 7:**
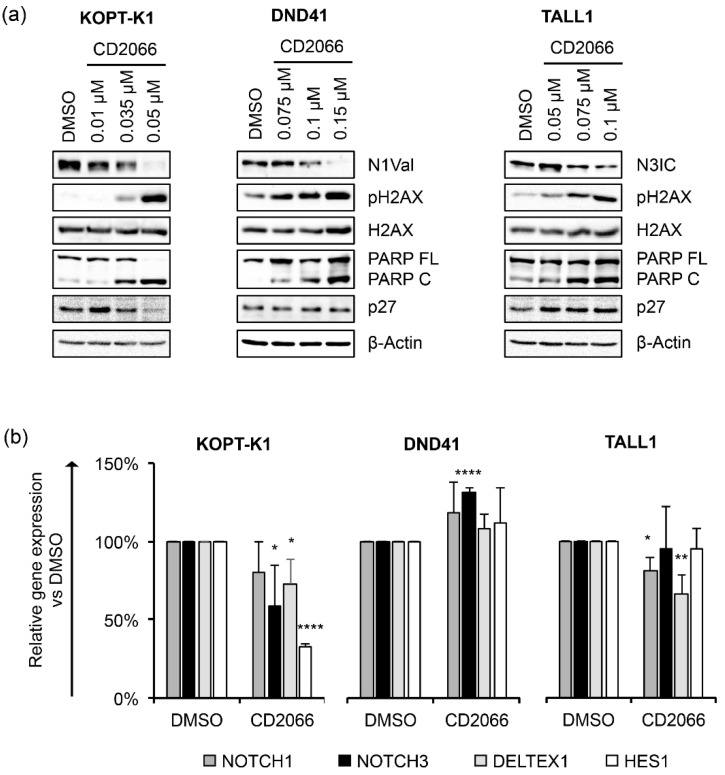
CD2066 counteracts Notch signaling and modulates cell cycle and apoptosis key factors in a dose-dependent manner in T-ALL cell lines. (**a**) Levels of N1Val, N3IC, pH2AX, H2AX, PARP FL, PARP C and p27 following 48 h of exposure to DMSO or CD2066 at the indicated concentrations. β-Actin was used as a loading control. Uncropped Western blots and the relative quantification of proteins levels related to this figure are displayed in Figure S9. (**b**) Relative NOTCH1, NOTCH3, DELTEX1, HES1 gene expression levels normalized to 18S rRNA after 48 h of exposure to CD2066 in KOPT-K1 (0.035 μM), DND41 (0.1 μM) and TALL1 (0.075 μM). Histograms show the mean of at least three independent experiments ± SD. Statistical significance was assessed using unpaired *t*-test. *p* values, * *p* < 0.05, ** *p* < 0.01, **** *p* < 0.0001.

Based on the observed anti-growth activity of CD2066, we focused on its mechanism of action by exploring whether, similarly with the scaffold of origin, its biological effects might combine with Notch signaling inhibition and DNA damage promotion. Therefore, using Western blotting, we determined the endogenous levels of N1Val, N3IC, pH2AX, PARP C and p27 in T-ALL cells following 48 h of exposure to doses around the IC_50_s. KOPT-K1 cells were treated with 0.01 μM, 0.035 μM, 0.05 μM of CD2066, DND41 cells were exposed to 0.075 μM, 0.1 μM, 0.15 μM of CD2066, and TALL1 cells were treated with 0.05 μM, 0.075 μM, 0.1 μM of CD2066. The results suggested that CD2066 might interfere with both Notch1 and Notch3 receptor activities and mediate DNA damage in T-ALL cells at low doses near the respective IC_50_s. Indeed, decreased N1Val and increased pH2AX were noticeable at a concentration of 0.035 μM in KOPT-K1 cells, while in DND41 N1Val, levels dropped at 0.1 μM and pH2AX rose at 0.075 μM. Similarly, in TALL1 cells, declined N3IC was observed at a concentration of 0.075 μM of CD2066, while pH2AX accumulation was detectable at dosages close to 0.05 μM (Figure 7a).

**Figure 8:**
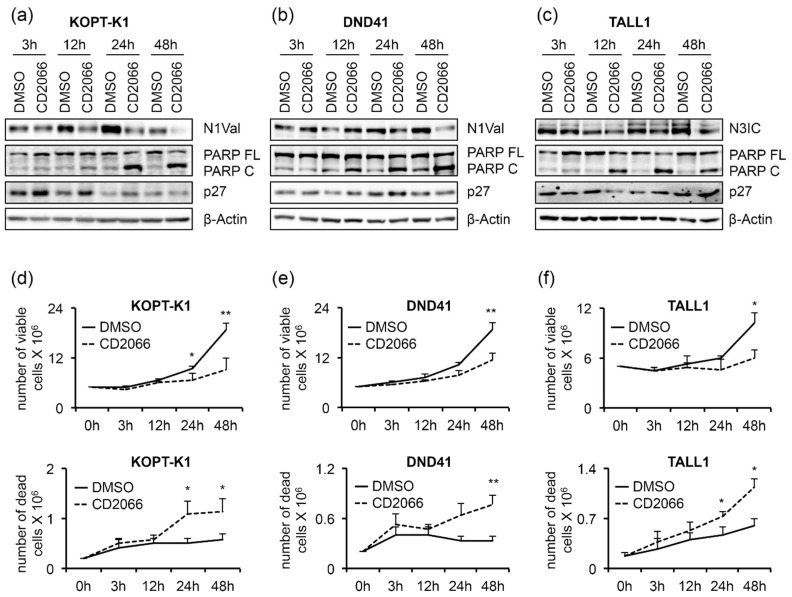
Decreased Notch activity precedes the antiviability effects of CD2066. T-ALL cells were treated for 48 h with DMSO or CD2066 at concentrations of 0.035 μM for KOPT-K1, 0.1 μM for DND41, and 0.075 μM for TALL1. Protein extracts were analyzed by Western blotting at 3 h, 12 h, 24 h, and 48 h. Protein expression levels of (**a**) N1Val in KOPT-K1 and (**b**) DND41, and (**c**) of N3IC in TALL1 cells. Levels of PARP FL, PARP C, and p27 in (**a**) KOPT-K1, (**b**) DND41, and (**c**) TALL1 cell lines at indicated time points. β–Actin was used as a loading control. Uncropped Western blots and the relative quantification of proteins levels related to this figure are displayed in Figure S12. The number of viable (upper panels) and dead (lower panels) (**d**) KOPT-K1, (**e**) DND41, and (**f**) TALL1 cells at different time points were evaluated through Trypan Blue exclusion assay. Data represent the mean value of three independent experiments ± SD. Statistical significance was calculated using a paired *t*-test. *p* values, * *p* < 0.05, ** *p* < 0.01.

Furthermore, exposure to the compound promoted PARP cleavage in a dose-dependent manner in all T-cells analyzed, while, intriguingly, it increased the levels of the cell-cycle regulator p27 only in TALL1-cells. Notch signaling suppression by CD2066 was further confirmed by decreased levels of the Notch transcriptional target genes NOTCH3, DELTEX1, and HES1 in KOPT-K1 and of NOTCH1 and DELTEX1 in TALL1 cells in response to 48 h of compound exposure. Unexpectedly, decreased N1Val following CD2066 treatment did not reflect the modulation of any Notch pathway-related genes we analyzed in DND41 cells (Figure 7b). However, the Notch1 target HES1 and the cytoplasmatic fragment of the Notch3 receptor were strongly downregulated at the protein level in DND41 cells upon 48 h of exposure to the compound, thus suggesting a post-transcriptional mechanism underlying CD2066-mediated Notch signaling regulation in this cell context (Figures S10 and S11). Furthermore, we detected decreased levels of N1Val and N3IC expression and accumulation of PARP C and p27, 12 h or 24 h after exposure to the compound of the above-described T-cells. Interestingly, molecular outcomes preceded or, at most, occurred simultaneously with, the associated antiviability and pro-death effects we observed after 24 h and 48 h of treatment, thus suggesting CD2066-mediated modulation of the above-mentioned pathways, including Notch, among its mechanisms of action (Figure 8a–f).

**Figure 9:**
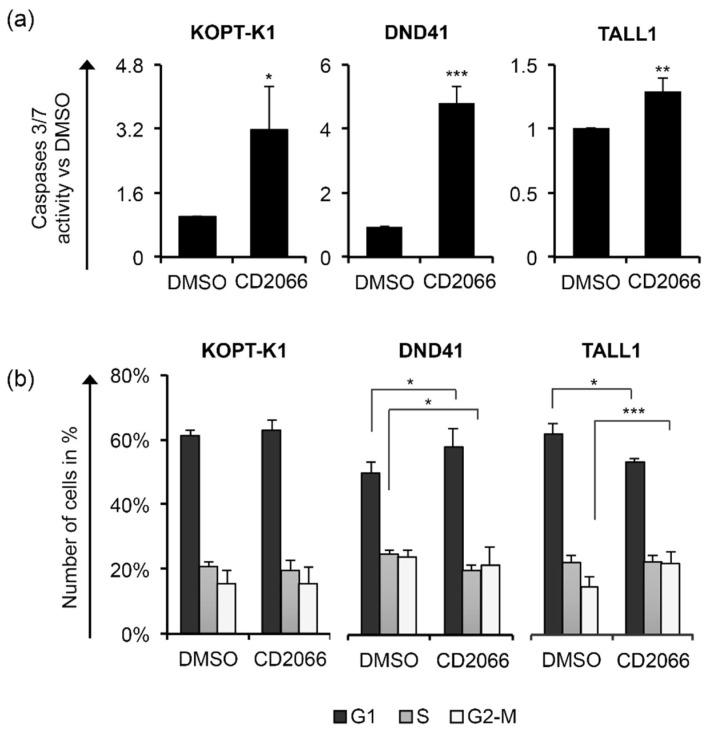
CD2066 treatment promotes apoptosis and/or corrupts cell-cycle progression in T-ALL. T-ALL cells were treated for 48 h with DMSO or CD2066 at a concentration of 0.035 μM for KOPT-K1, 0.1 μM for DND41, and 0.075 μM for TALL1. (**a**) Apoptosis was assessed by monitoring the levels of caspase 3 and 7 activity. (**b**) Cell distribution along cell-cycle phases was investigated by flow cytometry analysis of the DNA content after propidium iodide (PI) staining. The bars represent the mean of three independent experiments ± SD. Statistical significance was calculated using an unpaired *t*-test and paired *t*-test for caspase 3/7 activity and cell-cycle distribution, respectively. *p* values refer to the comparison between CD2066 and DMSO samples: * *p* < 0.05, ** *p* < 0.01, *** *p* < 0.001. Representative images of the cell-cycle analysis are shown in Figure S13.

According to the increased number of dead cells and PARP cleavage described above, the activities of caspases 3/7 were significantly upregulated by about three-fold in KOPT-K1, five-fold in DND41, and one-and-a-half-fold in TALL1 CD2066-treated cells compared to the controls, thereby confirming that cells underwent apoptosis (Figure 9a).

**Figure 10:**
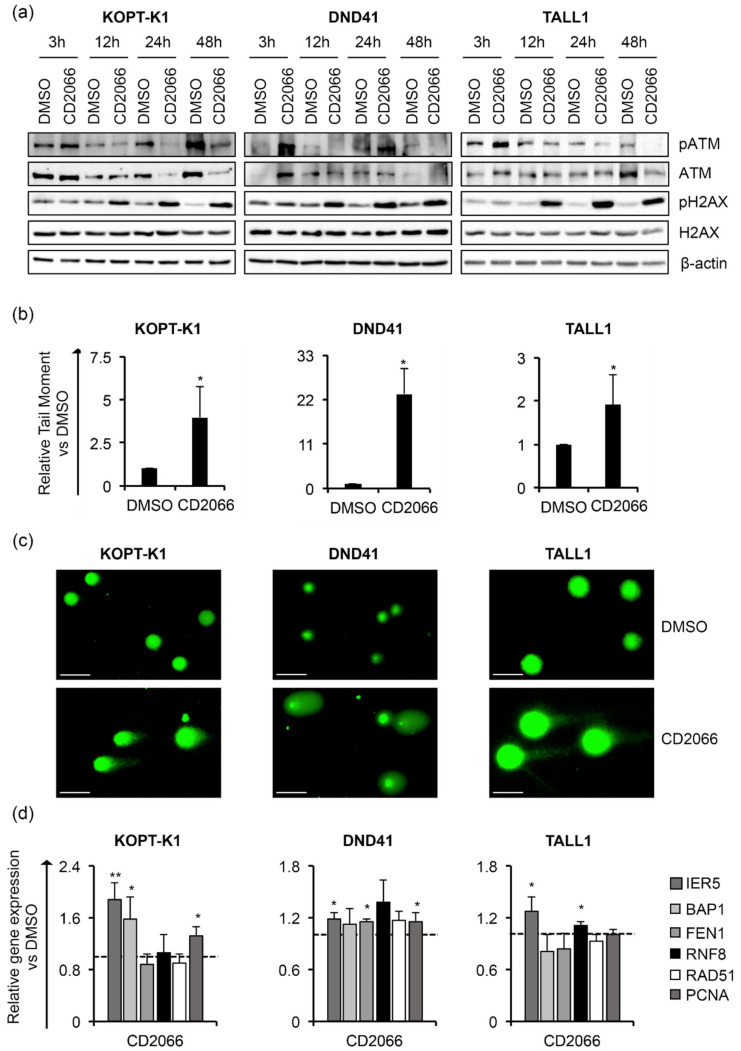
CD2066 promotes DNA damage in T-ALL cells. T-ALL cells were treated with CD2066 at the indicated concentrations: 0.035 μM for KOPT-K1, 0.1 μM for DND41, and 0.075 μM for TALL1. (**a**) Levels of pATM, ATM, pH2AX and H2AX in KOPT-K1, DND41 and TALL1 cells at indicated time points. β–actin was used as a loading control. Uncropped Western blots and the relative quantification of proteins levels related to this figure are displayed in Figure S14. (**b**) Relative quantification of the comet tail’s moment in KOPT-K1, DND41, and TALL1 evaluated at 24 h of treatment with CD2066. Data represent the mean value of three independent experiments ± SD. Statistical significance was calculated using an unpaired *t*-test, * *p* < 0.05. (**c**) Representative images of DNA comets in KOPT-K1, DND41, and TALL1 cells from CD2066’s treatment. Scale bar: 100 μm. (**d**) Relative transcriptional levels of IER5, BAP1, FEN1, RNF8, RAD51 and PCNA at 48 h of exposure to CD2066 versus DMSO. 18S rRNA was used as a loading control. Histograms show the mean results obtained from three independent experiments ± SD. Statistical significance was assessed using an unpaired *t*-test. *p* values, * *p* < 0.05, ** *p* < 0.01.

By FACS analysis after propidium iodide staining, we detected a slight cell-cycle arrest in the G1-phase in DND41 and in G2-M-phases in TALL1 cells after treatment with CD2066. In contrast, no interference with cell-cycle progression was observed in KOPT-K1 cells (Figure 9b). Altogether, these findings suggest that CD2066 is a novel compound with improved antiviability potential against T-ALL cells compared to curcumin. It corrupted Notch signaling and cell viability in T-cells at doses one hundred times lower than the starting compound (concentrations between 0.035 μM and 0.1 μM). Interestingly, as with curcumin, CD2066 treatment was associated with ATM activation, H2AX phosphorylation, and increased tail moments compared with the control cells, with tail moment increase ranging from about two- to twenty-three-fold (Figure 10a–c).

Further supporting DNA damage induction as a critical mechanism of action of CD2066 in T-ALL cells, the treatment promoted the gene expression of different regulators of the DNA-repair machinery, including IER5, BAP1 and PCNA in KOPT-K1, IER5, FEN1 and PCNA in DND41 cells and IER5 and RNF8 in TALL1 cells (Figure 10d). To assess CD2066 specificity for T-ALL and its prevalence for Notch modulation, we evaluated the effects of the treatment on Notch-independent Loucy T-ALL cells and U937 and THP1 AML cell lines (Figures S15 and S16) in which signaling has been suggested not to be essential for cell growth or promoting cell death, respectively [50]. Interestingly, CD2066 was less effective in these cell lines. The IC_50_s were about six, two and four times higher than the counterparts obtained in KOPT-K1, DND41 and TALL1, respectively, thus suggesting higher susceptibility of Notch-driven leukemic cells to the compound (Table 3 and Figure S17).

**Figure 11:**
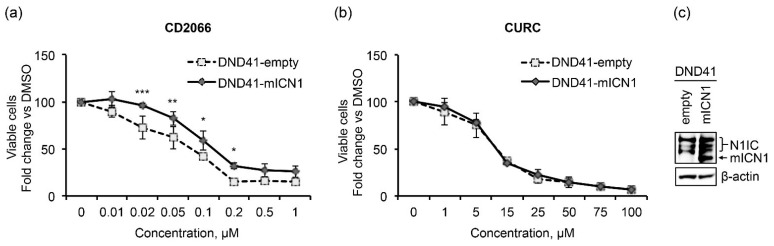
Notch1 signaling partially prevents the anti-growth effects of CD2066. Relative cell number in percentage evaluated by Trypan Blue exclusion assay of DND41 transduced with murine N1ICD (DND41-mICN1) and with the empty vector (DND41-empty) treated with CD2066 (**a**) and curcumin (**b**) at indicated concentrations. Data represent the mean value of triplicates normalized to the cell number of DMSO-treated cells at 48 h. SD is shown as error bars. Statistical significance was calculated using one way ANOVA followed by Sidak post hoc test. *p* values, * *p* < 0.05, ** *p* < 0.01, *** *p* < 0.001. (**c**) Levels of expression of endogenous (N1IC) and murine (mICN1) intracellular domain of Notch1 detected with an antibody against the C-terminal domain of the receptor in GFP-sorted DND41 cells transduced with mICN1 or with the empty control retroviruses. β-actin is used as a loading control. Uncropped Western blots and the relative quantification of proteins levels related to this figure are displayed in Figure S18.

We further investigated the link between Notch inhibition and antileukemic action of CD2066 by evaluating whether constitutive Notch signaling might hamper the antiviability effects of the compound. We found that the enforced activation of Notch1 partially protected DND41 cells transduced with a retrovirus encoding the murine N1IC (DND41-mICN1) cells from the anti-proliferative effects of CD2066 in the concentration range between 0.02 μM and 0.2 μM when compared with the relative empty retroviral vector-transduced cells (DND41-empty) (Figure 11a).

In contrast to CD2066, curcumin impacted the viability of DND41-empty and DND41-mICN1 cells with the same power of action (Figure 11b) and reduced proliferation in AML and Loucy cells similarly or to a greater extent than in T-ALL, thus suggesting lower specificity of action for T-ALL and Notch of the natural compound compared to its synthetic derivative (Table 4 and Figure S19). 

### 3.5. CD2066 Enhances KOPT-K1 Cell Line Sensitivity to CDK1 Inhibition

**Figure 12:**
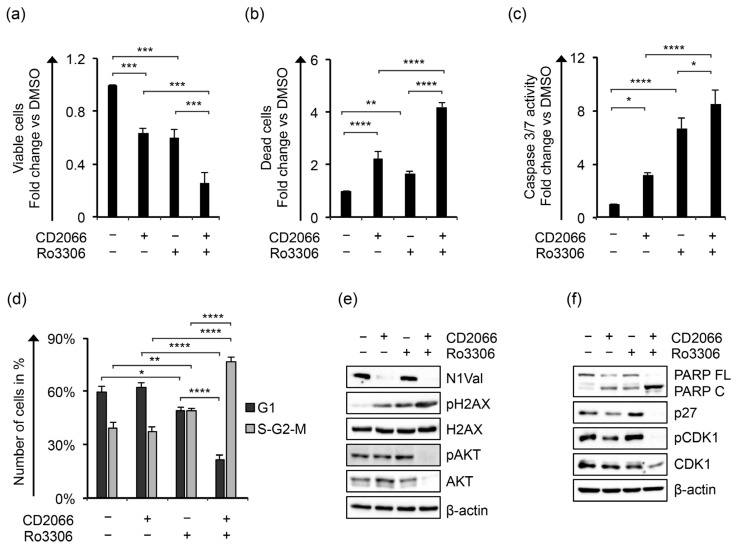
Ro3306 potentiates the antiviability effects of CD2066 in KOPT-K1 cells. The relative number of (**a**) viable and (**b**) dead cells, (**c**) levels of caspase 3 and 7 activities, (**d**) cell percentage in G1 versus S-G2-M phases, (**e**) protein expression levels of N1Val, pH2AX, H2AX, pAKT, AKT and (**f**) PARP FL, PARP C, p27, pCDK1, CDK1 in KOPT-K1 cells treated for 48 h with 0.035 μM of CD2066 and 2.5 μM of Ro3306 alone or in combination. The number of viable and dead cells was evaluated through Trypan Blue exclusion assay. Cell distribution between cell-cycle phases was analyzed by flow cytometry analysis of the DNA content after propidium iodide (PI) staining. Data represent the mean value of three independent experiments ± SD. Representative images of the cell-cycle analysis are shown in Figure S21. Statistical significance was calculated using one way ANOVA followed by Tukey post hoc test. *p* values, * *p* < 0.05, ** *p* < 0.01, *** *p* < 0.001, **** *p* < 0.0001. Uncropped Western blots and the relative quantification of proteins levels related to this figure are displayed in Figure S22.

Evidence has been produced that Notch signaling promoted cell cycle progression and proliferation in T-ALL by sustaining the activity of cyclin-dependent kinases (CDKs) [72] and that CDK inhibition halted T-ALL cell viability and survival in a xenograft model of this malignancy [73]. Recently, it has been shown that treatments with dinaciclib, a CDK1, CDK2, CDK5, and CDK9 inhibitor, arrested cell cycle progression in the G2/M phase and induced programmed cell death in T-ALL cells [74]. In particular, the kinases CDK1 and CDK2 were found to be among the top relevant kinases in a panel of T-ALL samples, and their inhibition by milciclib counteracted survival in cell lines and patient-derived xenografts of T-ALL [75], thus suggesting the targeting of these CDKs for pharmacological intervention in T-cell hematological malignancies. Consistent with this, we found that KOPT-K1 cells were highly sensitive to pharmacological inactivation of CDK1 by its commercially available inhibitor Ro3306, as 48 h of exposure to this drug decreased cell survival in a dose-dependent manner with an IC_50_ of 2.481 ± 0.332 μM (Figure S20). Interestingly, we found that cell exposure to 2.5 μM of Ro3306 decreased cell viability, increased cell death, promoted caspase 3/7 activation, and slightly halted cell-cycle progression in the S-G2/M phase, this being raised from about 39% ± 3% in control cells to 49% ± 1% in treated cells (Figure 12a–d). 

Notably, Ro3306 co-administration with CD2066 potentiated the anti-proliferative activity of the compounds compared to single treatment by enhancing the antiviability effects, the caspase-dependent apoptotic process, and causing a massive block of cell-cycle progression, reflected in 77% of the cell population being accumulated in the S-G2-M phase (Figure 12a–d). These features were further confirmed from a mechanistic point of view as the double treatment strongly enhanced the pro-apoptotic cleavage of PARP, decreased the activity of the pro-survival pathways of Notch1 and AKT, and reduced the expression of p27 and CDK1 when compared with single treatments (Figure 12e,f). In contrast to CD2066, Ro3306 alone promoted the expression of p27 and the inhibitory phosphorylation of CDK1 at tyrosine 15 (pCDK1). Moreover, Ro3306 induced the phosphorylation of AKT at serine 473 (pAKT), which was not affected by exposure to the curcuminoid and did not impact N1Val expression (Figure 12e). Of note, CD2066 and Ro3306 increased pH2AX levels similarly in single treatments, while combined treatment strongly enhanced it. Finally, assuming that the treatments acted by independent mechanisms, we analyzed their interaction using the Bliss independence model [76] in KOPT-K1 cells treated with a near-IC_50_ concentration of the compounds. By this approach, we found that CD2066 acted synergically with CDK1 inhibition, determined by the positive excess over Bliss value (EoB: 0.123 ± 0.022) and further indicated by the analysis of the combination index based on the Loewe additivity model (CI: 0.84 ± 0.01) [76].

## 4. Discussion

Thanks to the use of modern chemotherapy regimens, T-ALL is now considered a curable disease. Nevertheless, relapsed T-ALL shows unsatisfactory response rates to salvage chemotherapy and a dismal prognosis [77]. Importantly, unlike B-ALL, no targeted therapy has been approved for relapsed disease; therefore, developing novel therapies acting against signaling pathways that are critical for T-ALL pathogenesis represents a fundamental challenge for biomedical research [77,78]. Several Notch-interfering agents have been developed as therapeutic candidates for T-ALL and Notch-dependent cancers. These include the most extensively studied class of gamma-secretase inhibitors (GSI), which prevent Notch receptor cleavage/activation, and monoclonal blocking antibodies against distinct Notch receptors or their ligands. However, GSI treatment possesses substantial toxicity due to its interference with the Notch-dependent physiological maintenance of gastrointestinal homeostasis, impeding GSI dose escalation to therapeutically beneficial levels. On the other hand, although monoclonal antibodies are more selective than GSIs, they are not effective in contexts characterized by NOTCH receptor gene mutations [79]. Alternative approaches, such as epigenetic or post-translational modifiers, are at early preclinical stages of development and small molecules interfering with the function of the Notch transcription complex are emerging as more effective and potentially safer Notch inhibitors [18]. However, despite continuous efforts, no Notch-interfering treatment has been approved for clinical use, except for orphan drug approval for a few rare diseases [18].

In recent decades, naturally occurring compounds, such as curcumin, have been re-considered as promising candidates for the treatment of different pathologies, and several of these interfering with the expression of Notch signaling have been suggested as potentially safe anti-cancer agents [26]. Curcumin’s anti-tumor activity has been linked to the modulation of a wide range of pro-survival signaling pathways, such as nuclear factor-ĸB (NF-ĸB), p53, Wnt/β-catenin, phosphoinositide 3-kinase/protein kinase B (PI3-Kinase/AKT), activator protein 1 (AP-1), and mitogen-activated protein kinase (MAPK) pathways [80]. Interestingly, the anti-growth effects of curcumin and its derivatives are correlated with the repression of Notch1 signaling and its interacting pathways in some solid cancers, including osteosarcoma [81], prostate cancer [82], hepatocellular carcinoma [83], and glioblastoma [39], while in T-ALL, its anti-leukemic activity has been mainly linked to mitochondrial Ca2+ overload, reactive oxygen species (ROS) induction, and (PI3K)/AKT pathway inhibition, which lead to cell-cycle disruption and caspase-dependent apoptotic cell death.

Herein, we demonstrated that low doses of curcumin inhibited proliferation in T-ALL cell lines and that the cytotoxicity of curcumin was associated with decreased Notch pathway activity, irrespective of the cell proliferation dependence on distinct Notch receptors or genetic mutations. Notch signaling reduction has been confirmed both at the protein level of N1Val in KOPT-K1 and DND41 cells and of N3IC in TALL1 cells and in the transcriptional activation of NOTCH1 and/or NOTCH3 genes in all cell lines. Notch repression was further verified by attenuated expression of the direct target gene of the pathway DELTEX1. Curcumin exerted antiviability effects to a similar extent in the three cell lines, with IC_50_s in the concentration range between 6.3 μM and 13.3 μM, and it repressed N1Val and N3IC protein expression in a dose-dependent manner, starting from near-IC_50_ concentrations in distinct T-cells. Conversely, we focused on the DNA-damage-promoting capacity of this compound. Indeed, curcumin has demonstrated multiple effects on DNA damage and repair machinery in physiological conditions and experimental models of cancers [37,84]. Curcumin promoted the factors of base excision repair (BER) and non-homologous end-joining (NHEJ) pathways, thus preventing DNA damage accumulation and carcinogenesis in lymphocytes derived from healthy patients chronically exposed to arsenic [43]. However, it did not protect Jurkat T-lymphocytes and human peripheral blood mononuclear cells (PBMCs) from the DNA-damaging action of ROS and possibly induced DNA damage and cell death by favoring ROS generation [45,46]. Otherwise, curcumin counteracted DSB DDR through HDAC-inhibition and acetylation-mediated Rad52 inhibition [85]. An independent study showed that in Jurkat cells and resting human CD3+ T-cells, curcumin did not cause DNA damage or DDR pathway activation, even though it induced apoptosis [44]. However, in our hands, curcumin promoted the accumulation of the phosphorylated form of the DDR regulator ATM after 3 h of treatment and the levels of its target substrate pH2AX, starting from 12 h of treatment. It consistently caused increased DNA fragmentation, as revealed by comet assay at 24 h, and affected the expression of the DDR components FEN1, BRAT1, RAD51, and PCNA in a cell-line-dependent fashion indicating the DNA-damaging capacity of the molecule and its interference with DNA-repair machinery. The mechanisms of apoptosis induction and cell-cycle arrest mediated by curcumin have been widely investigated in several solid cancers and in AML cell lines, with contrasting results indicating possible cell-cycle arrest both in G1/S and G2/M phases [86,87,88,89,90,91,92,93]. In T-ALL, the only available evidence showed that 24 h of exposure to high concentrations of curcumin (40 μM) on MOLT4, CEM and Jurkat T-ALL cells interfered with the AKT pathway and promoted caspase-3 and PARP-1 cleavage activation-dependent apoptosis [41]. In our experimental setting, curcumin promoted the cleavage of poly-ADP ribose polymerase, enhanced the expression of the cell cycle regulator p27 and increased the activity of caspase 3 and 7 in all T-cells analyzed, and caused cell cycle block in the G2/M phase in DND41 cells, thus indicating that it acted mainly as a cytotoxic drug by inducing DNA damage and activating programmed cell death. Since curcumin exhibited antiviability effects in T-ALL cell lines in the low micromolar range, thus emerging as a promising basis for the design and synthesis of its novel derivatives endowed with enhanced anti-leukemic activity, we sought to improve its antiproliferative potential through additional modifications of its scaffold. Among seventeen curcumin derivatives generated by us through modification of the linker region between two aromatic rings and/or the addition of different functional groups in the benzene rings, the compound CD2066 showed strikingly potentiated antiviability activity in T-ALL cells at nanomolar concentrations. Furthermore, similarly to curcumin, it inhibited Notch1 and Notch3 receptor activation in a dose- and time-dependent manner in the near-IC_50_ concentration ranges starting from 24 h of treatment in KOPT-K1, TALL1, and DND41 cells, while, surprisingly, it led to a decreased transcriptional readout of Notch target genes in KOPT-K1 and TALL1 cells, but not in DND41 cells, in which the treatment reduced the protein levels of Notch1 targets Notch3 and HES1. These differences might be explained by different cell-context-dependent processes or by a specific molecular mechanism mediated by the compound CD2066 exerting different outcomes on Notch target genes through epigenetic modulations or protein stability regulation that were beyond the scope of our investigation. Similarly with curcumin, T-ALL cell exposure to the novel curcuminoid upregulated p27 expression and PARP cleavage and consistently promoted caspase 3/7 activity. Conversely, although the treatment resulted in DNA damage accumulation, it did not impinge on the repair machinery but, on the contrary, favored it. The treatment enhanced the DNA system components, as revealed by increased ATM phosphorylation associated with enhanced expression of IER5, BAP1, PCNA, FEN1, and RNF8. Moreover, CD2066 halted DND41 cell-cycle progression in the G1-phase and TALL1 in the G2-M-phase of the cell cycle, while it did not corrupt cell-cycle dynamics in KOPT-K1 cells. The lack of significant cell-cycle alterations, despite p27 upregulation in KOPT-K1 cells, in comparison with DND41 and TALL1, might be explained by a variable temporal sequence of these events in different cell lines or by the direct effects of the treatment on critical p27 interactors or downstream effectors in the specific cell line. Notably, Notch-dependent T-ALL cell lines were more susceptible to CD2066 action than the Notch-independent ETP-ALL cell line Loucy and AML cell lines U937 and THP1, as evidenced by an at least two-fold increase in IC_50_ values of the molecule in these cell lines. Moreover, exogenous expression of the murine N1IC domain partially rescued the antiviability effects of CD2066, thus suggesting Notch inhibition among the potential mechanisms underlying the biological effects of CD2066 in T-ALL cell models. Conversely, curcumin exerted anti-growth effects in Notch-independent cells to a similar extent to the Notch-dependent cell counterparts, thus indicating lower specificity of action for the signaling. 

Finally, to explore the feasibility of CD2066 application for chemotherapy-free drug combinations, we evaluated its effects in association with the inhibition of CDK1, recently found hyperactivated in T-ALL and, therefore, indicated as an appealing pharmacological target [74,75]. Our preliminary data on its combination with the commercially available CDK1 inhibitor Ro3306 revealed the compounds’ synergic antiviability effects associated with enhanced cell death, cell-cycle block in S-G2-M phases, and strong downregulation of pro-survival signals, such as pAKT in the KOPT-K1 cell line. Notably, CDK1 participates in DNA repair preventing replication-born DNA damage, and its pharmacological inhibition might upregulate the DNA damage hallmark pH2AX, as confirmed by our results, contributing to the lethal accumulation of double-strand breaks and providing a possible mechanistic explanation of the observed synergism [94]. Interestingly, Notch signaling participation in the DNA damage response has been evidenced on several levels. In TALL1 cells, Notch1 directly inhibited ATM kinase activity, thus contributing to the survival of Notch1-driven leukemias [95,96]. On the other hand, in the context of BRCA-deficient TNBC, Notch1 affected the DNA damage response in a pro-survival manner by enhancing the phosphorylation of ATR [97]. Otherwise, in FANCA-mutated Fanconi anemia characterized by pancytopenia and chromosomal instability due to dysregulated DNA repair, Notch1 overexpression facilitated defective hematopoietic cell proliferation [98]. Therefore, non-canonical participation of Notch in DNA-repair processes provides an inspiring mechanistic basis for parallel or consequent inhibition together with the application of DNA-damaging agents. The described effects of curcumin and its derivative with consideration of their double mode of action, and the association of strong Notch inhibitory and DNA-damaging capacity, together with strikingly low IC_50_ values in T-ALL cell lines, make CD2066 a promising anti-leukemic drug candidate.

## 5. Conclusions

We explored the antileukemic activity of curcumin in T-ALL cell lines based on its capacity to induce DNA damage and to downregulate Notch signaling activity. Moreover, we designed and synthesized a curcumin derivative, namely compound CD2066, endowed with potentiated antiproliferative activity in T-ALL cell lines in nanomolar concentrations. The scaffold molecule CD2066 antagonized Notch signaling, promoted DNA damage and synergized with the CDK1 inhibitor Ro3306, showing at least partial dependence of its antiviability effects on Notch signaling and relative specificity of its action for the T-ALL context. The above-described spectrum of CD2066 effects makes it a promising candidate for development as an antileukemic agent for T-ALL treatment.

## Figures and Tables

**Table 1:** Curcumin inhibits cell viability in T-ALL cell lines. IC_50_ values of curcumin were determined in KOPT-K1, DND41, and TALL1 cell lines. Data represent mean values of three independent experiments ± standard deviation of the mean (SD).

Cell Line	IC_50_ Value ± SD, μM
KOPT-K1	8.220 ± 0.817
DND41	13.255 ± 2.269
TALL1	6.330 ± 0.884

**Table 2:** CD2066 is a potent curcumin-based compound that inhibits T-ALL cell viability. IC_50_s of the curcumin derivatives CD2066, CD2067 and EC109 determined after 48 h of treatment with growing doses of the compounds in indicated cell lines. The number of viable cells was measured by Trypan Blue assay. Data represent the mean value of at least three independent experiments ± SD.

Compound	Cell Line	IC_50_ Value ± SD, μM
CD2066	KOPT-K1 TALL1	0.032 ± 0.006 0.060 ± 0.008
CD2067	KOPT-K1 TALL1	1.974 ± 0.622 2.290 ± 0.274
EC109	KOPT-K1 TALL1	7.757 ± 0.027 1.471 ± 0.128

**Table 3:** Loucy, U937 and THP1 cells are more resistant to CD2066 than KOPT-K1, DND41 and TALL1 cell lines. IC_50_ values of CD2066 were evaluated in Loucy, U937 and THP1 cell lines after 48 h of treatment. Data represent the mean values of three independent experiments ± SD of the mean.

Cell Line	IC_50_ Value ± SD, μM
Loucy	0.233 ± 0.012
U937	0.218 ± 0.034
THP1	0.201 ± 0.024

**Table 4:** Curcumin affects cell viability in Notch-independent cells similarly to Notch-dependent cells. IC_50_ values of curcumin were evaluated in Loucy, U937, and THP1 cell lines after 48 h of treatment. Data represent the mean values of three independent experiments ± SD.

Cell Line	IC_50_ Value ± SD, μM
Loucy	6.851 ± 0.871
U937	3.854 ± 0.409
THP1	7.447 ± 1.025

## Data Availability

The data presented in this study are available in the article and the Supplementary Materials.

## Supplementary Materials

The following supporting information can be downloaded at: https://www.mdpi.com/article/10.3390/cancers14235772/s1, Figure S1: Curcumin inhibits T-ALL cell growth. Dose-response curves by Trypan blue cell viability assay towards KOPT-K1, DND41, and TALL1 cells at 48 h incubation times with curcumin. Data represent the mean values of three independent experiments ± SD; Figure S2: Uncropped Western blots related to main Figure 1a and relative quantification of proteins levels. The expression of each protein was analyzed by densitometry and presented in the figure as a ratio to the control sample (DMSO) normalized to β-actin (RQ). Other samples (OS); Figure S3: Uncropped Western blots related to main Figure 2a–c and relative quantification of proteins levels. The expression of each protein was analyzed by densitometry and presented in the figure as a ratio to the control sample (DMSO) normalized to β-actin (RQ). Other samples (OS); Figure S4: Impact of curcumin on the cell cycle progression in T-ALL cells. KOPT-K1, DND41 and TALL1 cells were treated for 48 h with curcumin or vehicle alone (DMSO) with the doses indicated in the main text. Cell cycles were investigated by flow cytometry analysis of DNA content after propidium iodide (PI) staining. FACS plots shown in the figure are representative of results obtained from three independent experiments; Figure S5: Uncropped Western blots related to main Figure 4a and relative quantification of proteins levels. The expression of each protein was analyzed by densitometry and presented in the figure as a ratio to the control sample (DMSO) normalized to β-actin (RQ); Figure S6: Library of curcumin derivatives; Figure S7: CD2066 is more potent than CD2067 and EC109 in terms of antiviability activity. Dose-response curves by Trypan blue cell viability assay towards KOPT-K1 and TALL1 cells at 48 h incubation times with CD2066, CD2067 and EC109. Data represent the mean values of at least three independent experiments ± SD; Figure S8: CD2066 inhibits DND41 cell growth. Dose-response curves by Trypan blue cell viability assay towards DND41 cells at 48 h incubation times with CD2066. Data represent the mean values of three independent experiments ± SD; Figure S9: Uncropped Western blots related to main Figure 7a and relative quantification of proteins levels. The expression of each protein was analyzed by densitometry and presented in the figure as a ratio to the control sample (DMSO) normalized to β-actin (RQ). Other samples (OS); Figure S10: CD2066 reduces N3IC and HES1 protein expression in DND41 cells. Levels of N3IC and HES1 following 48 h of exposure to DMSO or CD2066 at the indicated concentrations. β-Actin was used as a loading control. Uncropped western blots and relative quantification of proteins levels related to this figure are displayed in Figure S11; Figure S11: Uncropped Western blots related to Figure S10 and relative quantification of proteins levels. The expression of each protein was analyzed by densitometry and presented in the figure as a ratio to the control sample (DMSO) normalized to β-actin (RQ). Other samples (OS); Figure S12: Uncropped Western blots related to main Figure 8a–c and relative quantification of proteins levels. The expression of each protein was analyzed by densitometry and presented in the figure as a ratio to the control sample (DMSO) normalized to β-actin (RQ). Other samples (OS); Figure S13: CD2066 effects on cell cycle progression in T-ALL cell lines. KOPT-K1, DND41 and TALL1 cells were treated for 48 h with CD2066 or vehicle alone (DMSO) with the doses indicated in the main text. Cell cycles were investigated by flow cytometry analysis of DNA content after Propidium iodide (PI) staining. FACS plots shown in the figure are representative of results obtained from three independent experiments; Figure S14: Uncropped Western blots related to main Figure 10a and relative quantification of proteins levels. The expression of each protein was analyzed by densitometry and presented in the figure as a ratio to the control sample (DMSO) normalized to β-actin (RQ). Other samples (OS); Figure S15: Notch signaling activity in distinct leukemia cell contexts. Levels of basal expression of N1Val, N1IC, N3IC, and HES1 in KOPT-K1, DND41, TALL1, Loucy, U937, and THP1 cells. β-Actin was used as a loading control. Uncropped western blots and relative quantification of proteins levels related to this figure are displayed in Figure S16; Figure S16. Uncropped Western blots related to Figure S15 and relative quantification of proteins. The expression of each protein was analyzed by densitometry and presented in the figure as values normalized to β-actin (OD). Other samples (OS); Figure S17: CD2066 antiviability effects on Notch-deficient leukemia cells. Dose-response curves by Trypan blue cell viability assay towards Loucy, U937, and THP1 cells at 48 h incubation times with CD2066. Data represent the mean values of three independent experiments ± SD. Figure S18: Uncropped Western blots related to main Figure 11c and relative quantification of proteins levels. The expression of each protein was analyzed by densitometry and presented in the figure as values normalized to β-actin (OD). Other samples (OS); Figure S19: Curcumin antiviability effects on Notch-deficient leukemia cells. Dose-response curves by Trypan blue cell viability assay towards Loucy, U937, and THP1 cells at 48 h incubation times with curcumin. Data represent the mean values of three independent experiments ± SD; Figure S20: Ro3306 inhibits KOPT-K1 cell viability. Dose-response curves by Trypan blue cell viability assay towards KOPT-K1 cells at 48 h incubation times with Ro3306. Data represent the mean values of three independent experiments ± SD; Figure S21: Effects of single and combined treatment with CD2066 and Ro3306 in KOPT-K1 cell cycle progression. KOPT-K1 cells were treated for 48 h with CD2066 and Ro3306 alone or in combination with the doses indicated in the main text. Cell cycles were investigated by flow cytometry analysis of DNA content after Propidium iodide (PI) staining. FACS plots shown in the figure are representative of results obtained from three independent experiments; Figure S22: Uncropped Western blots related to main Figure 12e,f and relative quantification of proteins. The expression of each protein was analyzed by densitometry and presented in the figure as a ratio to the control sample (DMSO) normalized to β-actin (RQ). Other samples (OS); Table S1. Sequences and annealing temperatures of primers for real-time RT-PCR.
