# Supplementary material for: Identification of a Novel Curcumin Derivative Influencing Notch Pathway and DNA Damage as a Potential Therapeutic Agent in T-ALL
Source: Cancers (Basel). 2022 Nov 24;14(23):5772. doi: 10.3390/cancers14235772 (PMC9736653; doi:10.3390/cancers14235772)
Supplement: Supplementary file 1 [file cancers-14-05772-s001.zip › cancers-2024722-supplementary.pdf]

SUPPLEMENTARY MATERIAL

LIST OF THE SUPPLEMENTARY MATERIAL:

SUPPLEMENTARY FIGURES

Figure S1. Curcumin inhibits T-ALL cell growth;
Figure S2. Uncropped Western Blots related to main figure 1a and relative quantification of proteins levels;
Figure S3. Uncropped Western Blots related to main figure 2a–c and relative quantification of proteins levels;
Figure S4. Impact of curcumin on the cell cycle progression in T-ALL cells;
Figure S5. Uncropped Western Blots related to main figure 4a and relative quantification of proteins levels;
Figure S6. Library of curcumin derivatives;
Figure S7. CD2066 is more potent than CD2067 and EC109 in terms of antiviability activity;
Figure S8. CD2066 inhibits DND41 cell growth;
Figure S9. Uncropped Western Blots related to main figure 7a and relative quantification of proteins levels;
Figure S10. CD2066 reduces N3IC and HES1 protein expression in DND41 cells;
Figure S11. Uncropped Western Blots related to figure S10 and relative quantification of proteins levels;
Figure S12. Uncropped Western Blots related to main figure 8a-c and relative quantification of proteins levels;
Figure S13. CD2066 effects on cell cycle progression in T-ALL cell lines;
Figure S14. Uncropped Western Blots related to main figure 10a and relative quantification of proteins levels;
Figure S15. Notch signaling activity in distinct leukemia cell contexts;
Figure S16. Uncropped Western Blots related to figure S15 and relative quantification of proteins;
Figure S17. CD2066 antiviability effects on Notch-deficient leukemia cells;
Figure S18. Uncropped Western Blots related to main figure 11c and relative quantification of proteins levels;
Figure S19. Curcumin antiviability effects on Notch-deficient leukemia cells;
Figure S20. Ro3306 inhibits KOPT-K1 cell viability;
Figure S21. Effects of single and combined treatment with CD2066 and Ro3306 in KOPT-K1 cell cycle progression;
Figure S22. Uncropped Western Blots related to main figure 12e-f and relative quantification of proteins.

SUPPLEMENTARY TABLES

Table S1. Sequences and annealing temperatures of primers for real-time RT-PCR.

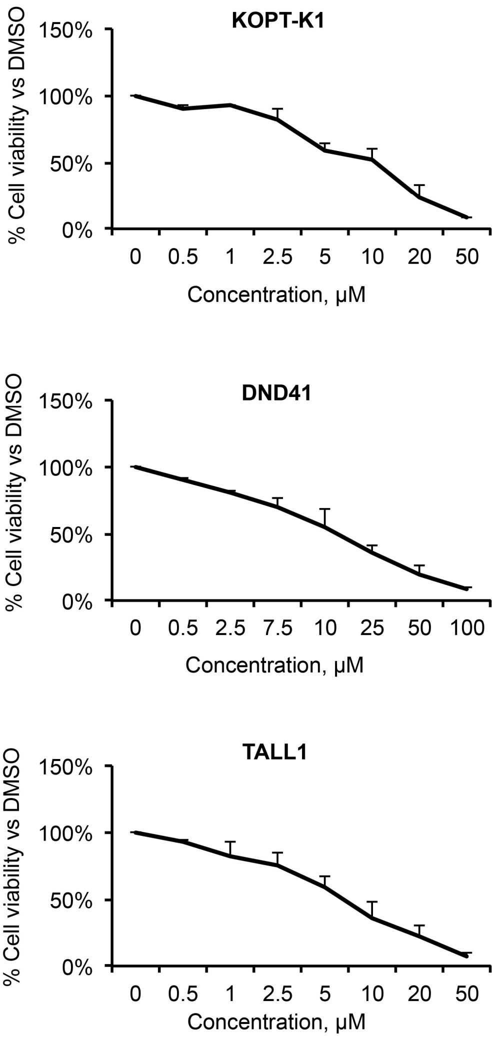

Figure S1. Curcumin inhibits T-ALL cell growth. Dose-response curves by Trypan blue cell viability assay towards KOPT-K1, DND41, and TALL1 cells at 48 h incubation times with curcumin. Data represent the mean values of three independent experiments ± SD.

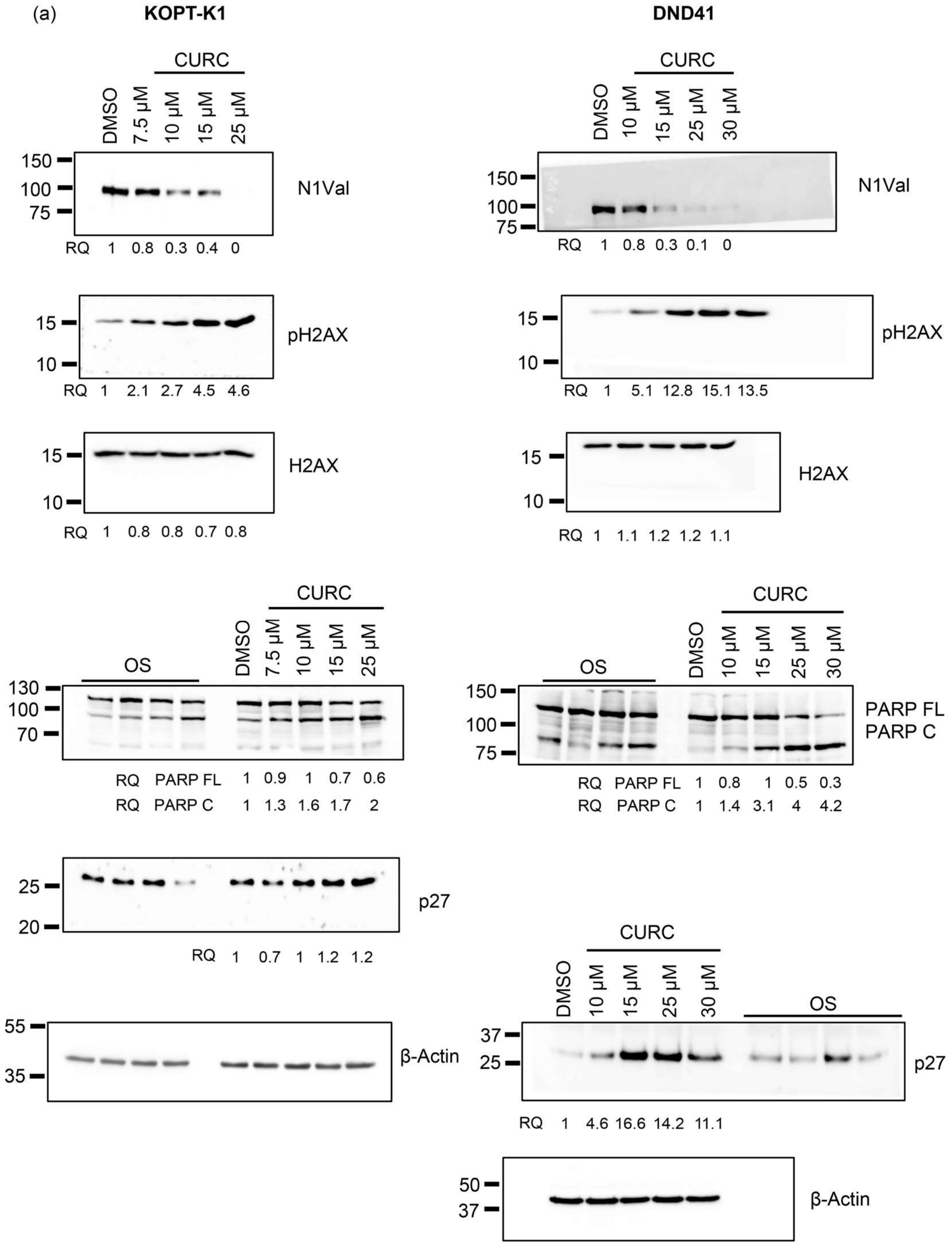

Figure continued on next page

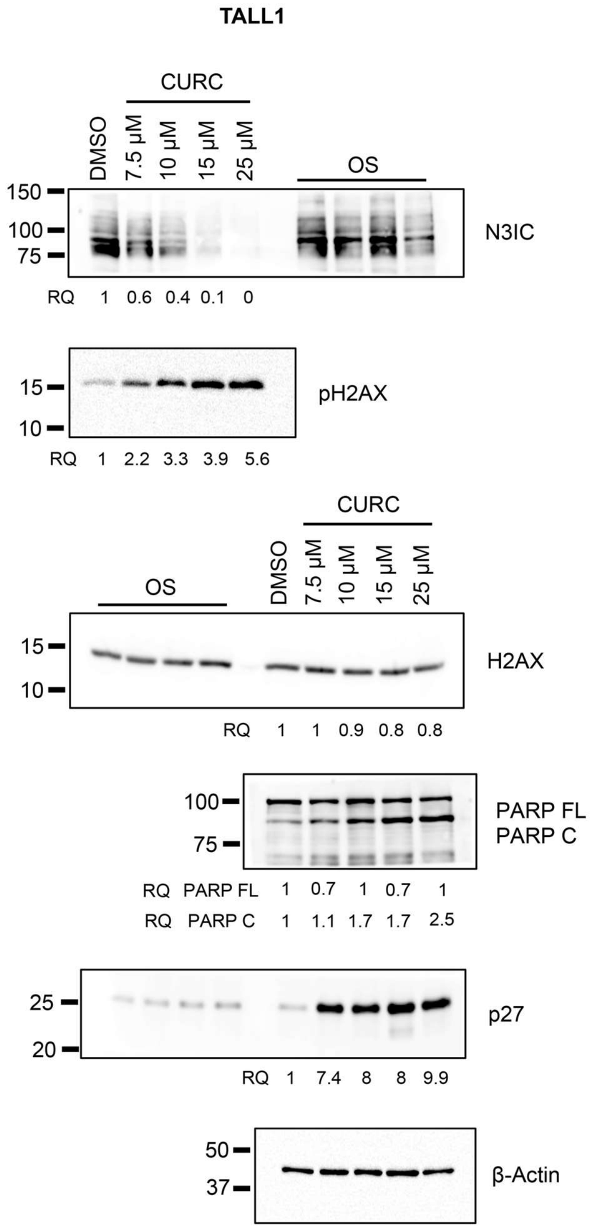

Figure S2. Uncropped Western Blots related to main Figure 1a and relative quantification of proteins levels. The expression of each protein was analyzed by densitometry and presented in the figure as a ratio to the control sample (DMSO) normalized to β-actin (RQ). Other samples (OS).

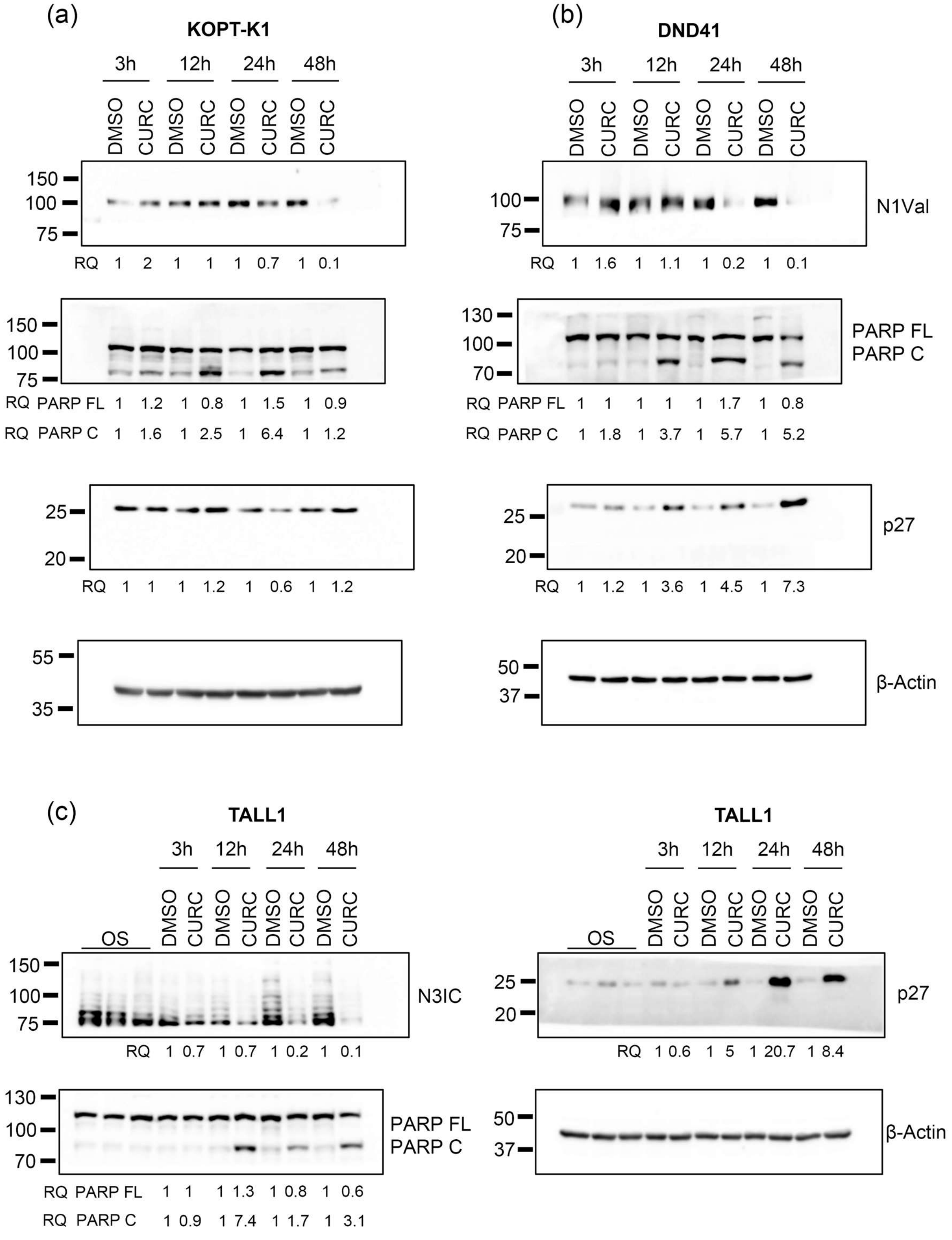

Figure S3. Uncropped Western Blots related to main Figure 2a-c and relative quantification of protein levels . The expression of each protein was analyzed by densitometry and presented in the figure as a ratio to the control sample (DMSO) normalized to β-actin (RQ). Other samples (OS).

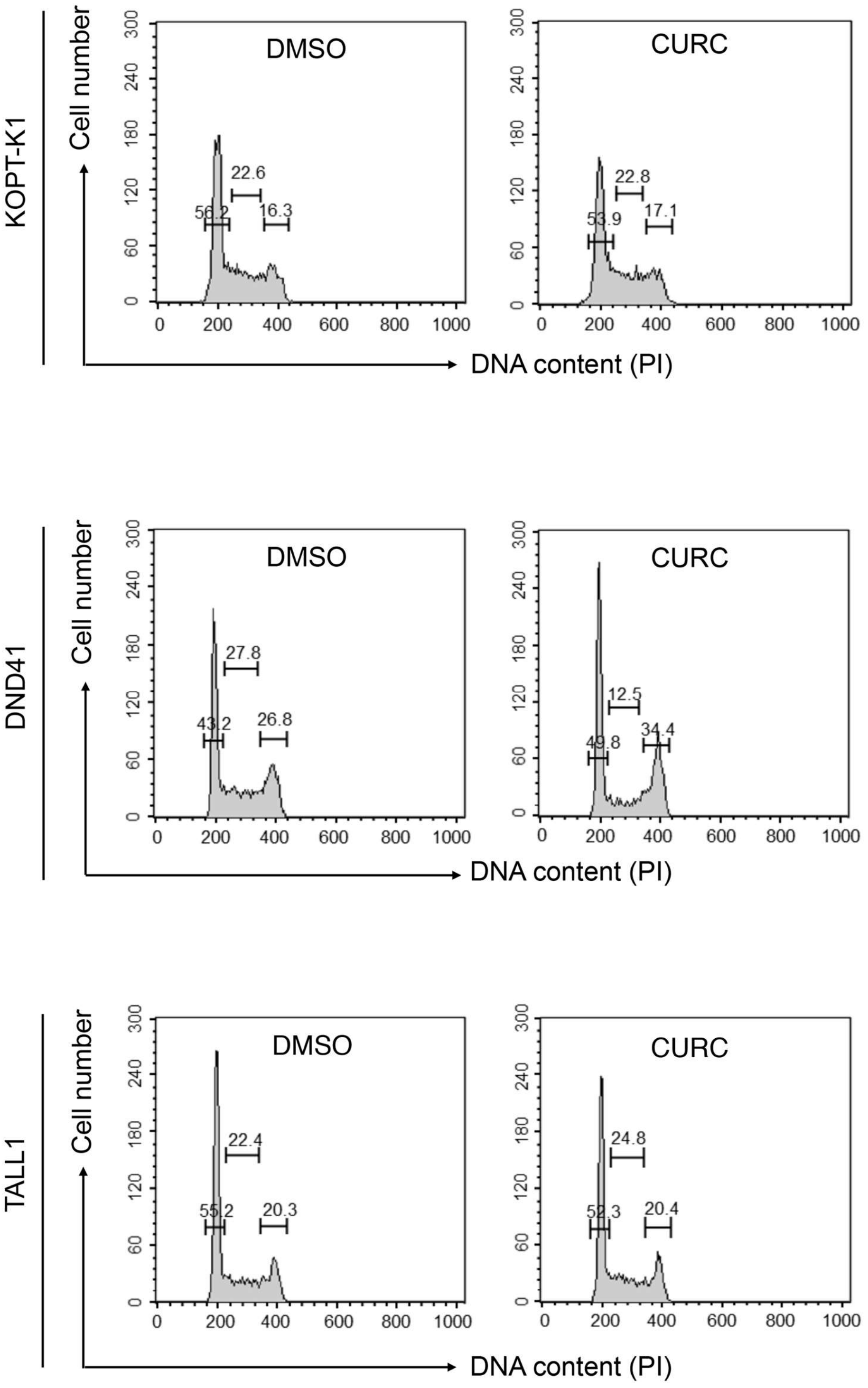

Figure S4. Impact of curcumin on the cell cycle progression in T-ALL cells. KOPT-K1, DND41 and TALL1 cells were treated for 48 h with curcumin or vehicle alone (DMSO) with the doses indicated in the main text. Cell cycles were investigated by flow cytometry analysis of DNA content after propidium iodide (PI) staining. FACS plots shown in the figure are representative of results obtained from three independent experiments.

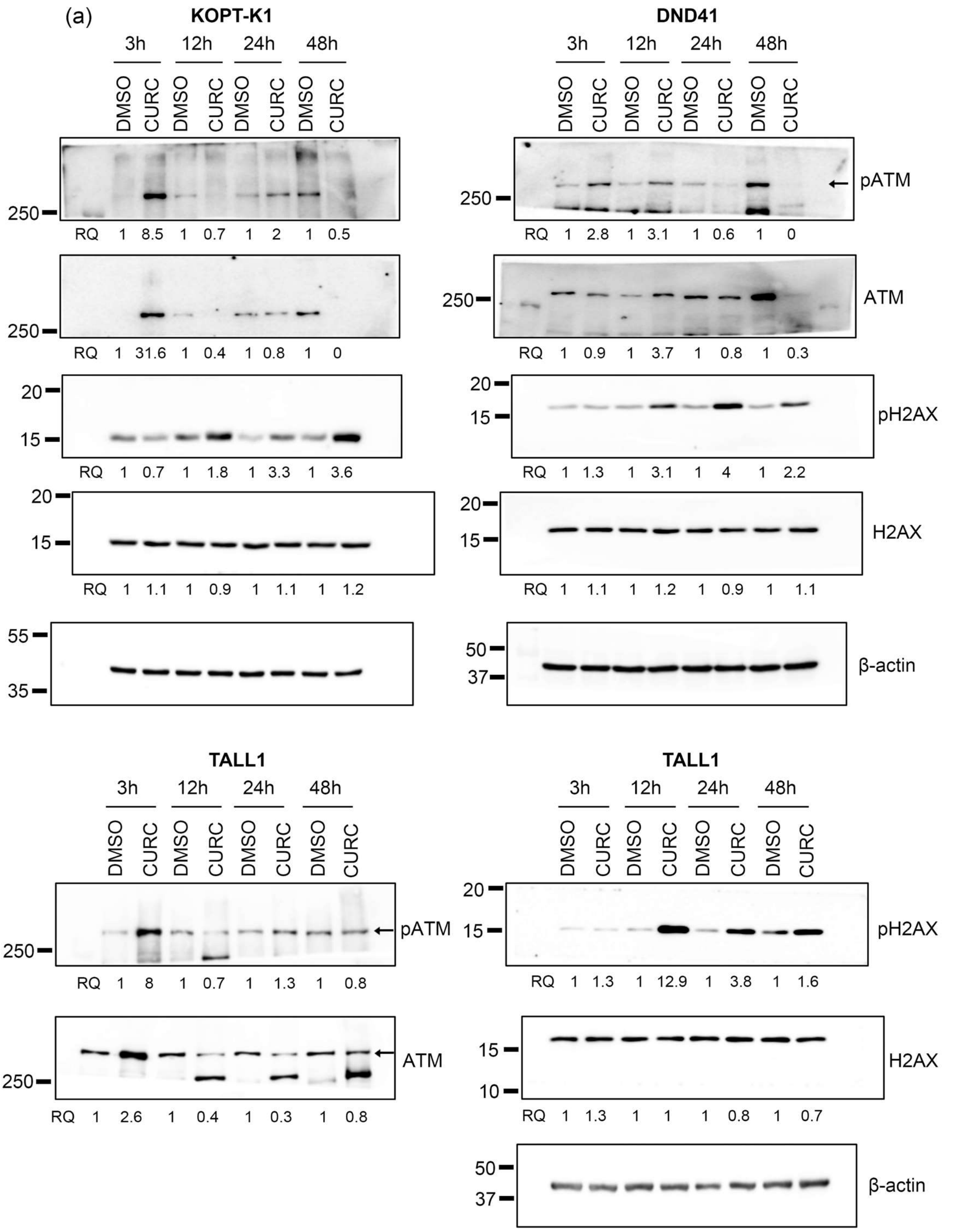

Figure S5. Uncropped Western Blots related to main figure 4a and relative quantification of proteins levels. The expression of each protein was analyzed by densitometry and presented in the figure as a ratio to the control sample (DMSO) normalized to β-actin (RQ).

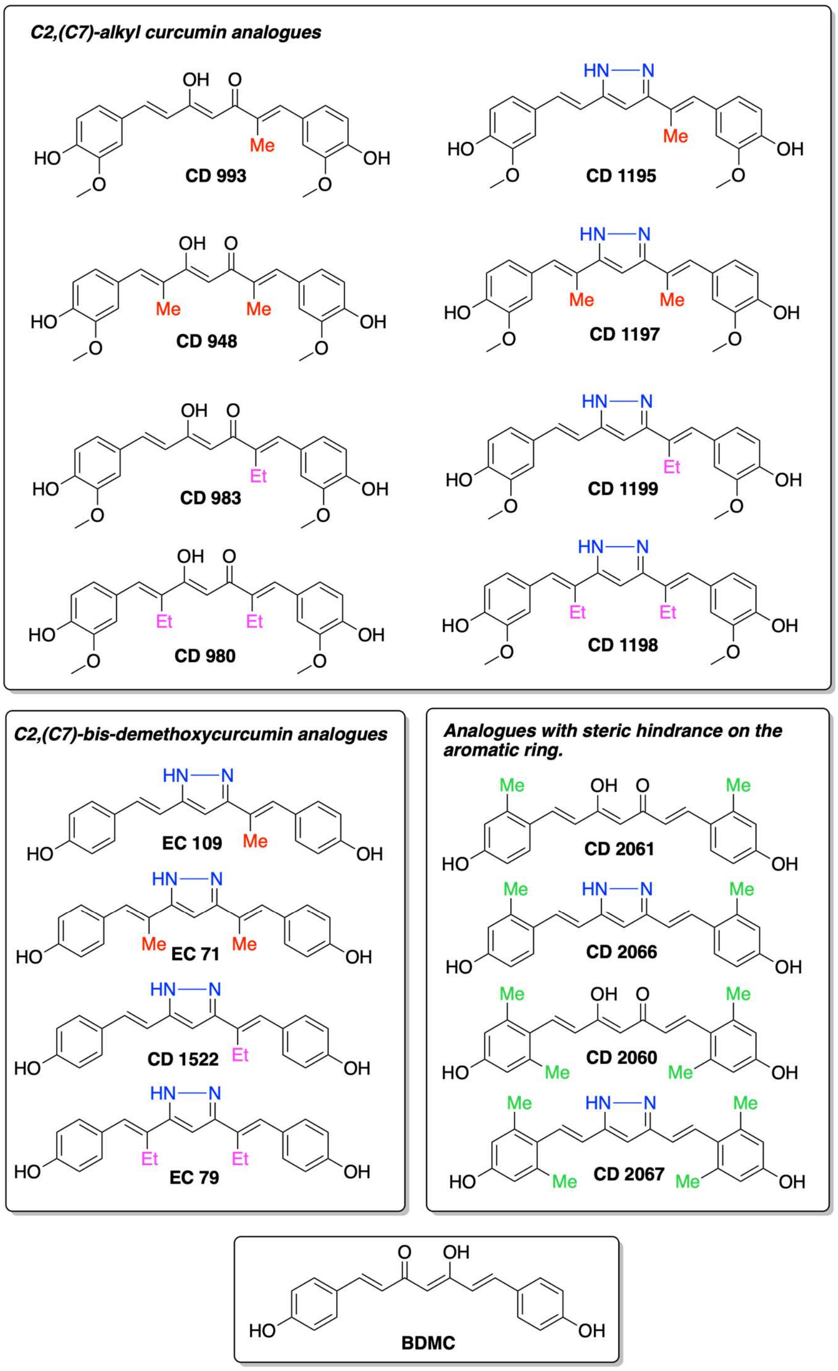

Figure S6. Library of curcumin derivatives.

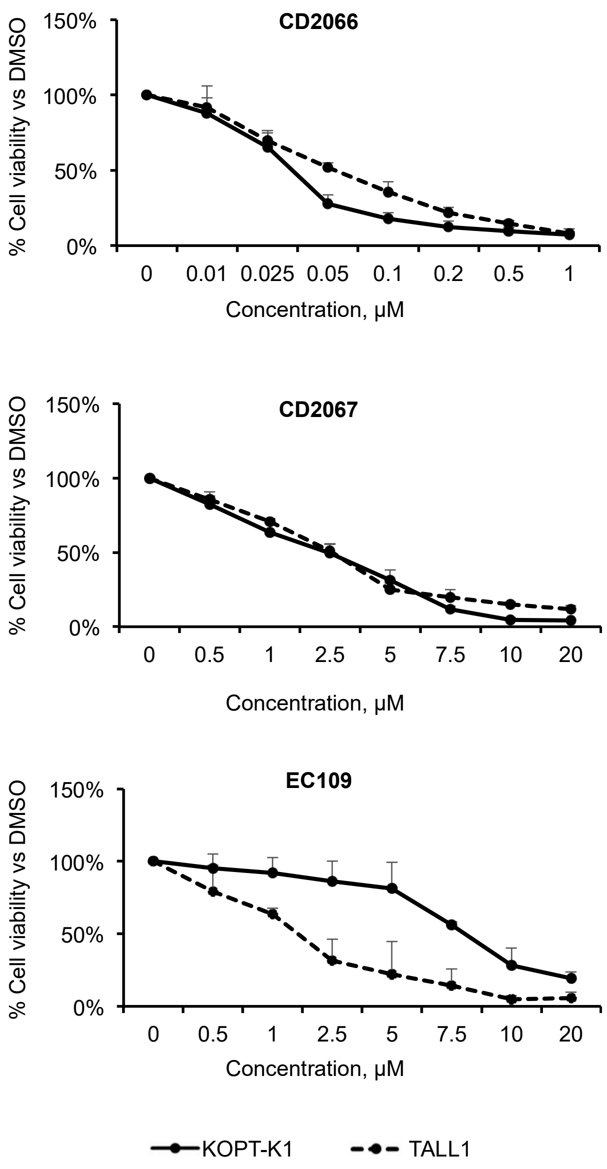

Figure S7. CD2066 is more potent than CD2067 and EC109 in terms of antiviability activity. Dose-response curves by Trypan blue cell viability assay towards KOPT-K1 and TALL1 cells at 48 h incubation times with CD2066, CD2067 and EC109. Data represent the mean values of at least three independent experiments ± SD.

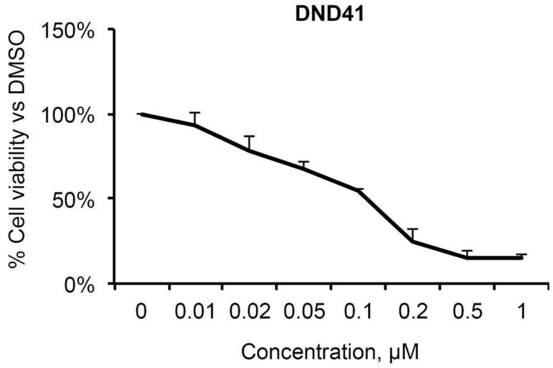

Figure S8. CD2066 inhibits DND41 cell growth. Dose-response curves by Trypan blue cell viability assay towards DND41 cells at 48 h incubation times with CD2066. Data represent the mean values of three independent experiments ± SD.

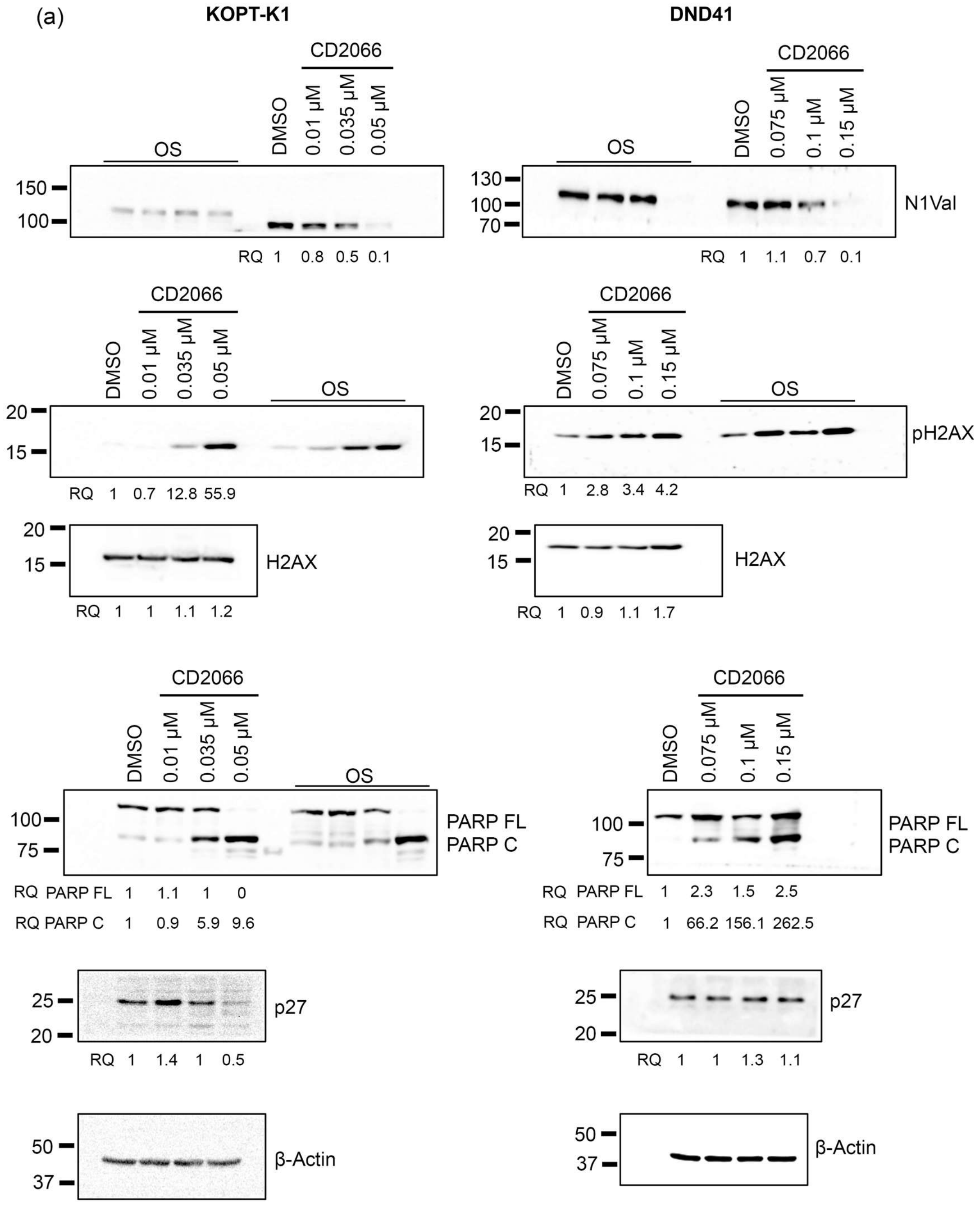

Figure continued on next page

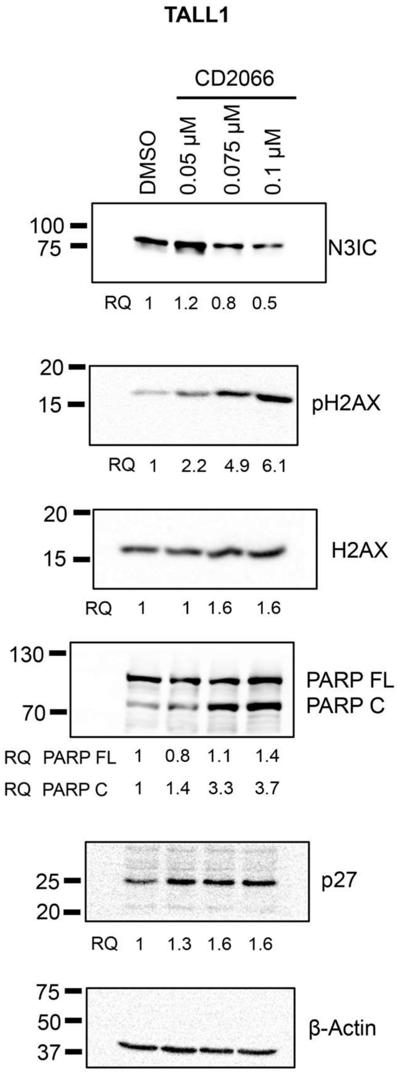

Figure S9. Uncropped Western Blots related to main figure 7a and relative quantification of proteins levels. The expression of each protein was analyzed by densitometry and presented in the figure as a ratio to the control sample (DMSO) normalized to β-actin (RQ). Other samples (OS).

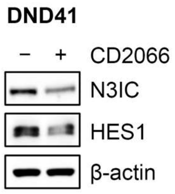

Figure S10. CD2066 reduces N3IC and HES1 protein expression in DND41 cells. Levels of N3IC and HES1 following 48 h of exposure to DMSO or CD2066 at the indicated concentrations. β-Actin was used as a loading control. Uncropped western blots and relative quantification of proteins levels related to this figure are displayed in Figure S11.

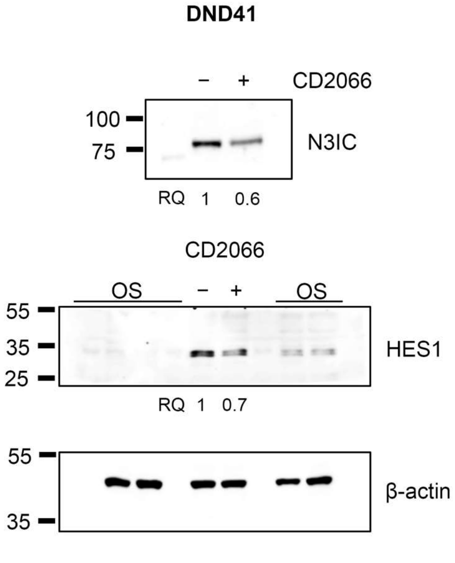

Figure S11. Uncropped Western Blots related to Figure S10 and relative quantification of proteins levels. The expression of each protein was analyzed by densitometry and presented in the figure as a ratio to the control sample (DMSO) normalized to β-actin (RQ). Other samples (OS).

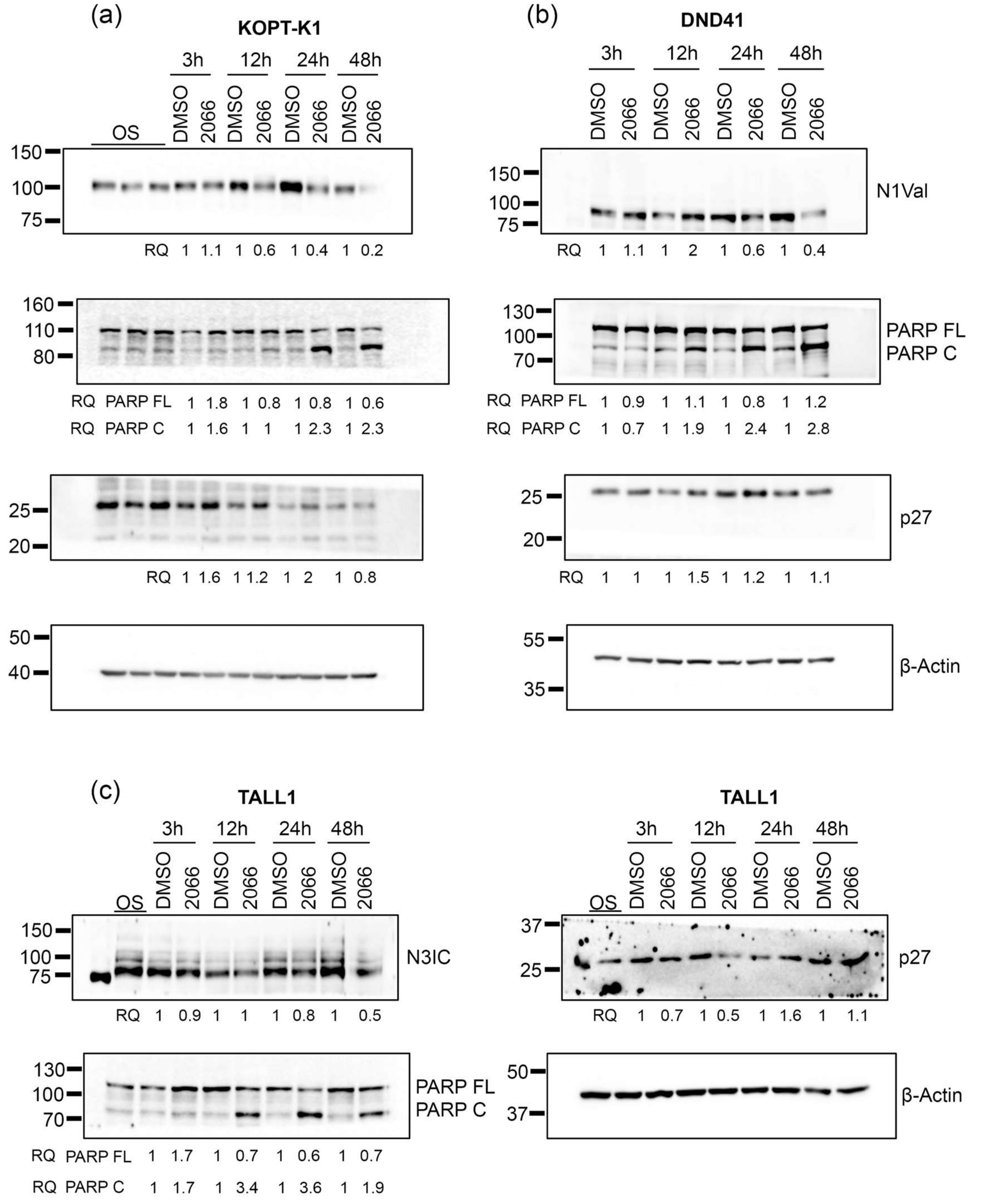

Figure S12. Uncropped Western Blots related to main figure 8a-c and relative quantification of proteins levels. The expression of each protein was analyzed by densitometry and presented in the figure as a ratio to the control sample (DMSO) normalized to β-actin (RQ). Other samples (OS).

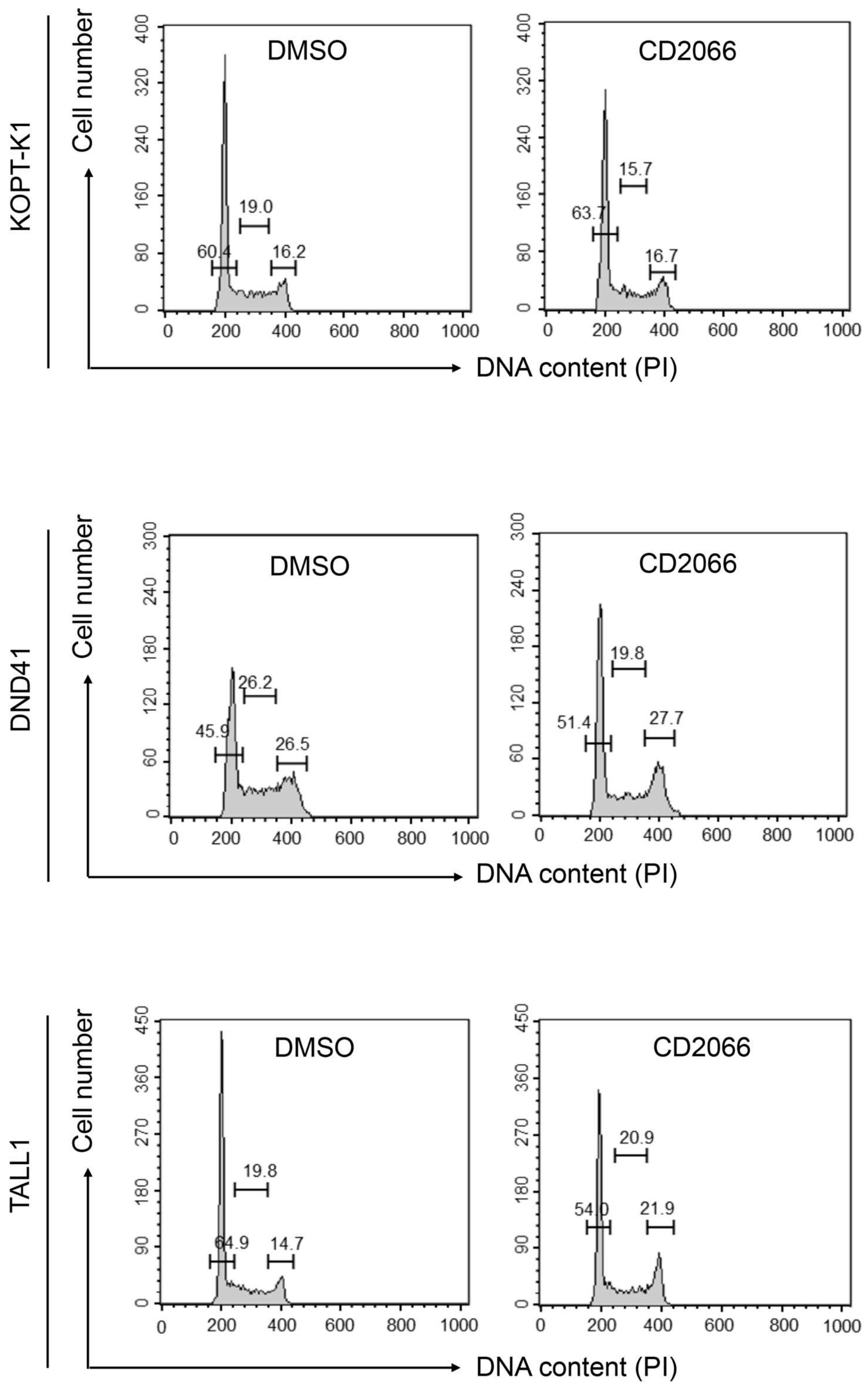

Figure S13. CD2066 effects on cell cycle progression in T-ALL cell lines. KOPT-K1, DND41 and TALL1 cells were treated for 48 h with CD2066 or vehicle alone (DMSO) with the doses indicated in the main text. Cell cycles were investigated by flow cytometry analysis of DNA content after Propidium iodide (PI) staining. FACS plots shown in the figure are representative of results obtained from three independent experiments.

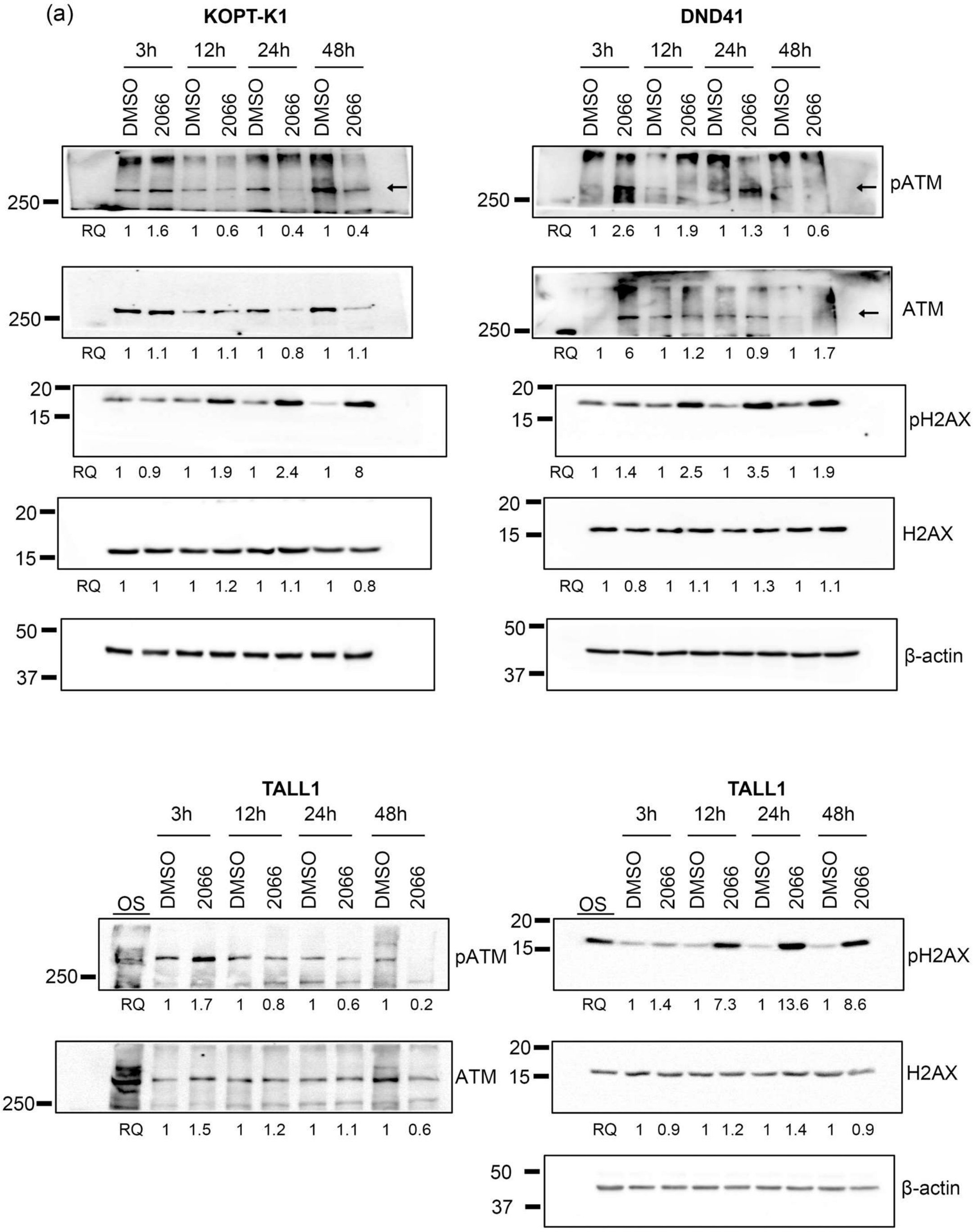

Figure S14. Uncropped Western Blots related to main figure 10a and relative quantification of proteins levels. The expression of each protein was analyzed by densitometry and presented in the figure as a ratio to the control sample (DMSO) normalized to β-actin (RQ). Other samples (OS).

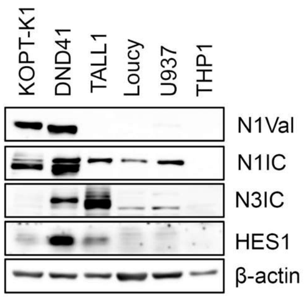

Figure S15. Notch signaling activity in distinct leukemia cell contexts. Levels of basal expression of N1Val, N1IC, N3IC, and HES1 in KOPT-K1, DND41, TALL1, Loucy, U937, and THP1 cells. β-Actin was used as a loading control. Uncropped western blots and relative quantification of proteins levels related to this figure are displayed in Figure S16.

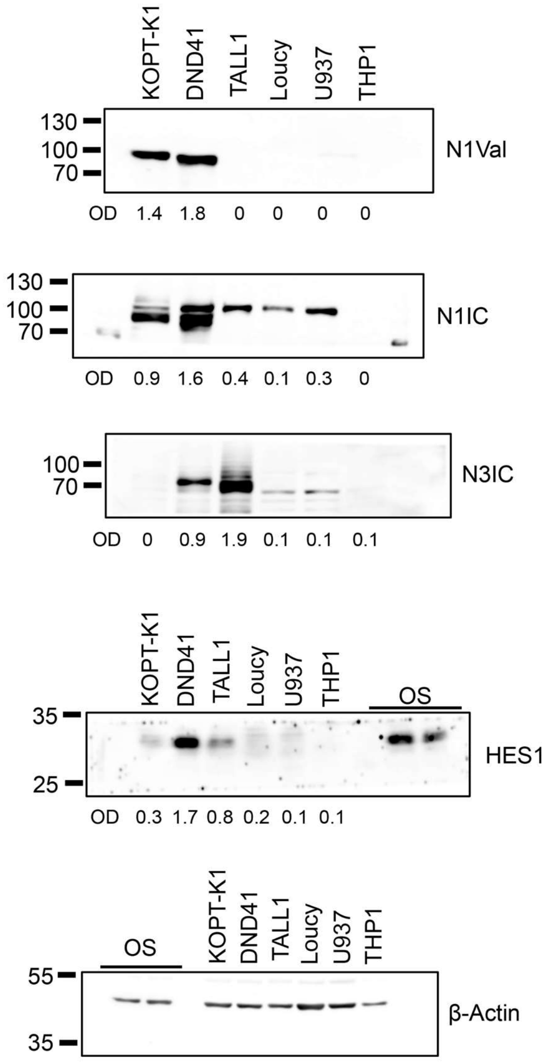

Figure S16. Uncropped Western Blots related to Figure S15 and relative quantification of proteins. The expression of each protein was analyzed by densitometry and presented in the figure as values normalized to β-actin (OD). Other samples (OS).

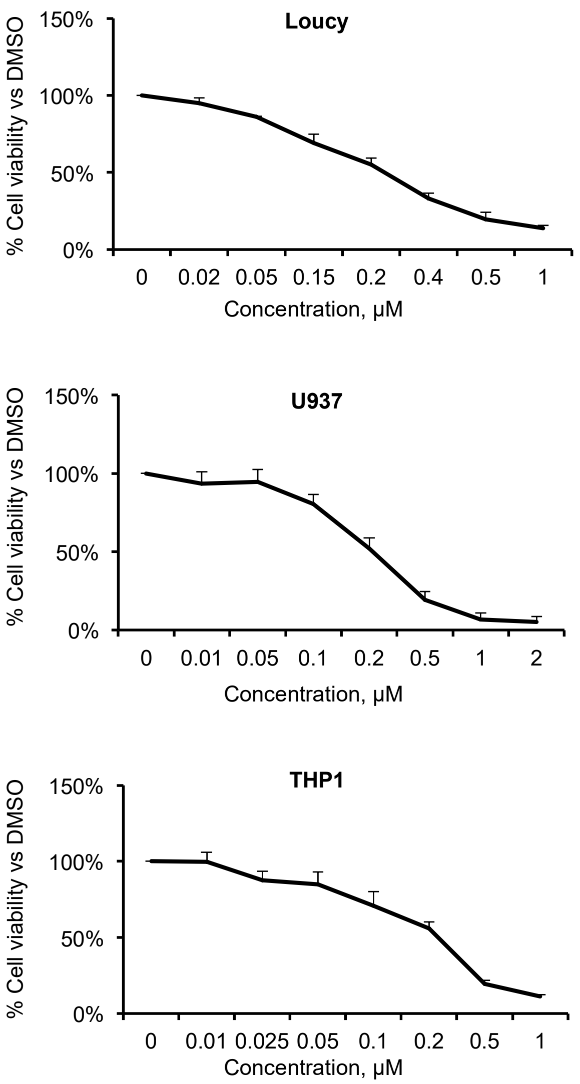

Figure S17. CD2066 antiviability effects on Notch-deficient leukemia cells. Dose-response curves by Trypan blue cell viability assay towards Loucy, U937, and THP1 cells at 48 h incubation times with CD2066. Data represent the mean values of three independent experiments ± SD.

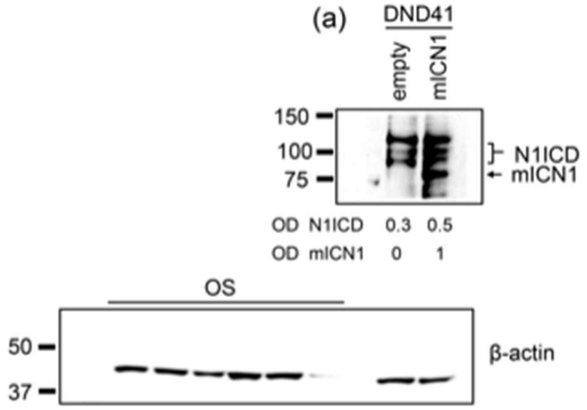

Figure S18. Uncropped Western Blots related to main Figure 11c and relative quantification of proteins levels. The expression of each protein was analyzed by densitometry and presented in the figure as values normalized to β-actin (OD). Other samples (OS).

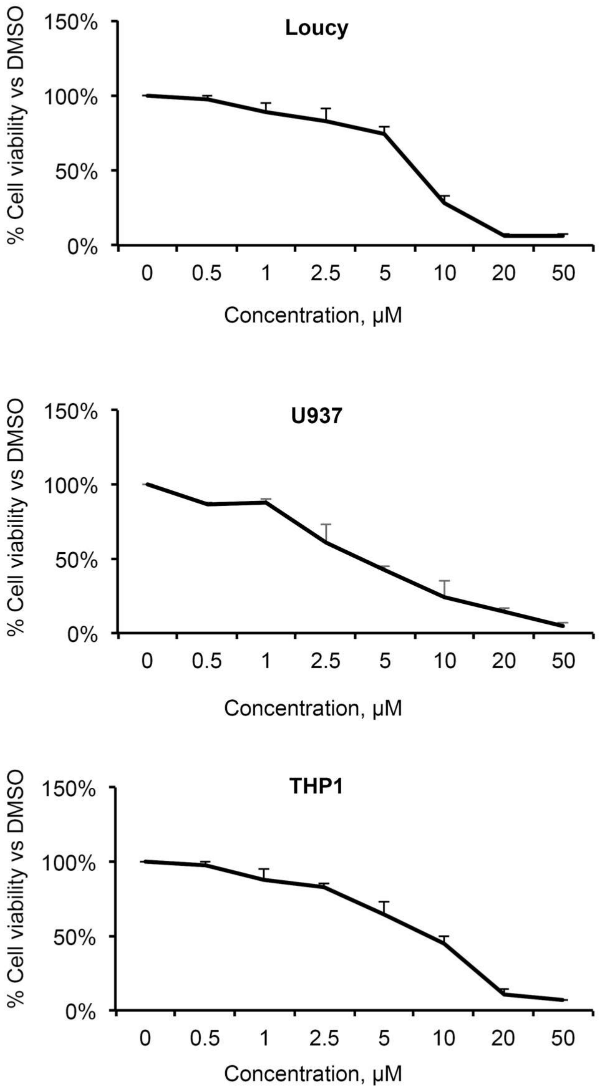

Figure S19. Curcumin antiviability effects on Notch-deficient leukemia cells. Dose-response curves by Trypan blue cell viability assay towards Loucy, U937, and THP1 cells at 48 h incubation times with curcumin. Data represent the mean values of three independent experiments ± SD.

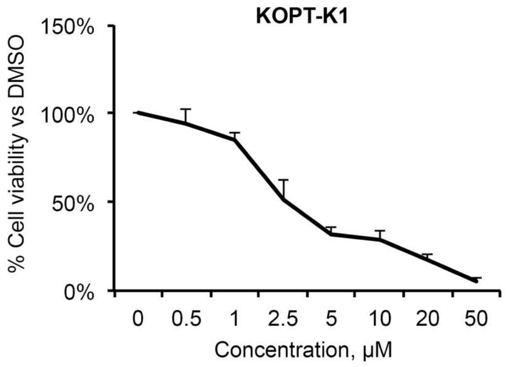

Figure S20. Ro3306 inhibits KOPT-K1 cell viability. Dose-response curves by Trypan blue cell viability assay towards KOPT-K1 cells at 48 h incubation times with Ro3306. Data represent the mean values of three independent experiments ± SD.

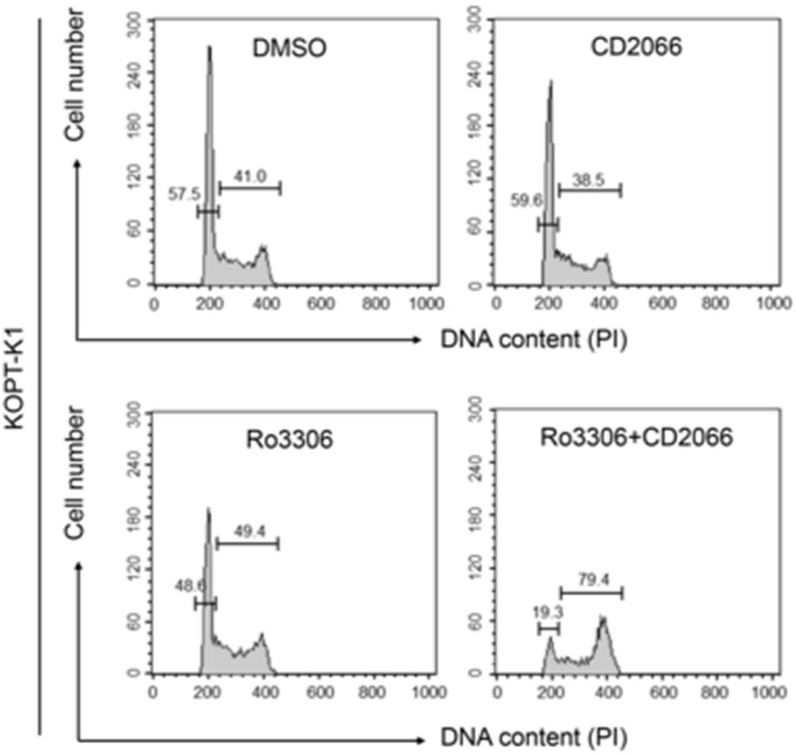

Figure S21. Effects of single and combined treatment with CD2066 and Ro3306 in KOPT-K1 cell cycle progression. KOPT-K1 cells were treated for 48 h with CD2066 and Ro3306 alone or in combination with the doses indicated in the main text. Cell cycles were investigated by flow cytometry analysis of DNA content after Propidium iodide (PI) staining. FACS plots shown in the figure are representative of results obtained from three independent experiments.

(e)

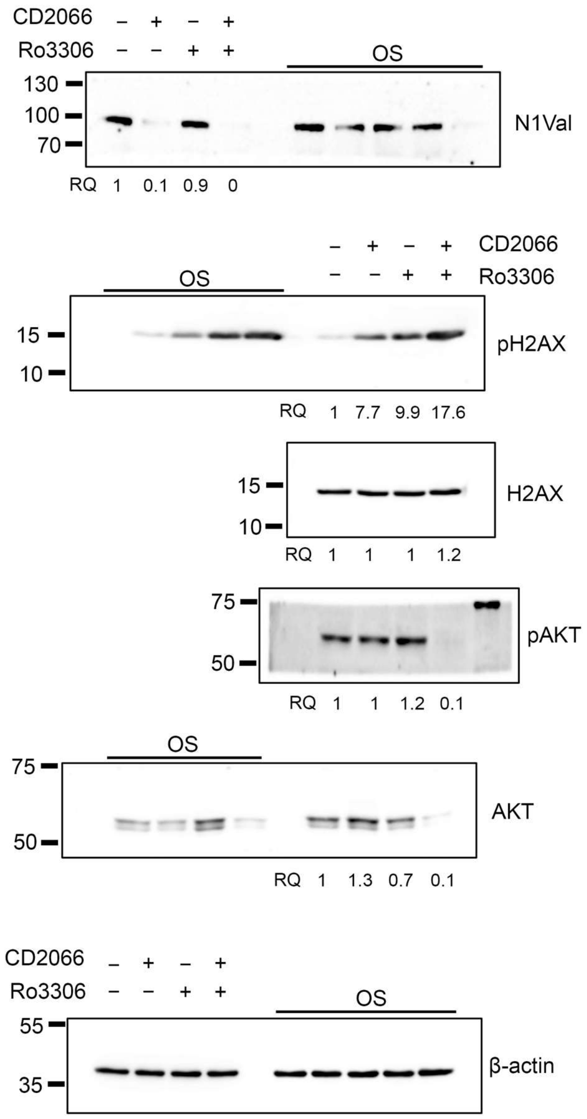

Figure continued on next page

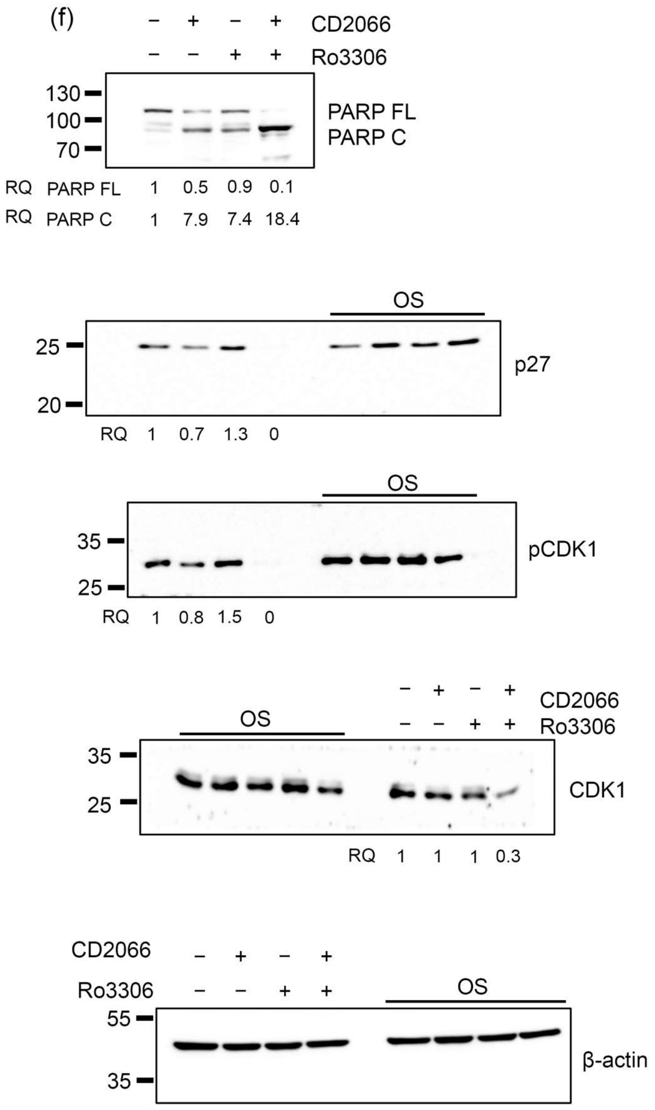

Figure S22. Uncropped Western Blots related to main Figure 12 e,f and relative quantification of proteins. The expression of each protein was analyzed by densitometry and presented in the figure as a ratio to the control sample (DMSO) normalized to β-actin (RQ). Other samples (OS).

Table S1. Sequences and annealing temperatures of primers for real-time RT-PCR.

Gene Name	Primer Name	Sequences 5′- 3′	Annealing temperature
IER5	IER5-Forward	GACTCCTACGGAAAAGCCCC	60°
	IER5-Reverse	CTCTCAGCACCGGCTTATCG	60°
BAP1	BAP1-Forward	AGAGTACAGACACGGCCTCT	60°
	BAP1-Reverse	AGCAGCTCCTTGGGTGAGTAT	60°
FEN1	FEN1-Forward	GCTACCGAGGACATGGACTG	60°
	FEN1-Reverse	TTCATTTGGCTCGCTCCACT	60°
RNF8	RNF8-Forward	AAGATGGGTGCGAGGTGACT	60°
	RNF8-Reverse	ACTGGCCACTTCACCTTTCC	60°
RAD51	RAD51-Forward	ATACTGTGGAGGCTGTTGCC	60°
	RAD51-Reverse	AGTTGCAGTGGTGAAACCCA	60°
PCNA	PCNA-Forward	CTGAGGGCTTCGACACCTAC	60°
	PCNA-Reverse	CGCGTTATCTTCGGCCCTTA	60°
18S	18S-Forward	CACGCCAGTACAAGATCCGA	60°
	18S-Reverse	TTCACGGAGCTTGTTGTCCA	60°
